# Indium(II) Chloride as a Precursor in the Synthesis of Ternary (Ag–In–S) and Quaternary (Ag–In–Zn–S) Nanocrystals

**DOI:** 10.1021/acs.chemmater.1c03800

**Published:** 2022-01-03

**Authors:** Patrycja Kowalik, Piotr Bujak, Mateusz Penkala, Anna M. Maroń, Andrzej Ostrowski, Angelika Kmita, Marta Gajewska, Wojciech Lisowski, Janusz W. Sobczak, Adam Pron

**Affiliations:** † Faculty of Chemistry, Warsaw University of Technology, Noakowskiego 3, 00-664 Warsaw, Poland; ‡ Faculty of Chemistry, University of Warsaw, Pasteura 1 Street, PL-02-093 Warsaw, Poland; § Institute of Chemistry, Faculty of Science and Technology, University of Silesia, Szkolna 9, 40-007 Katowice, Poland; ∥ Academic Centre for Materials and Nanotechnology, AGH University of Science and Technology, al. Mickiewicza 30, 30-059 Krakow, Poland; ⊥ Institute of Physical Chemistry, Polish Academy of Science, Kasprzaka 44/52, 01-224 Warsaw, Poland

## Abstract

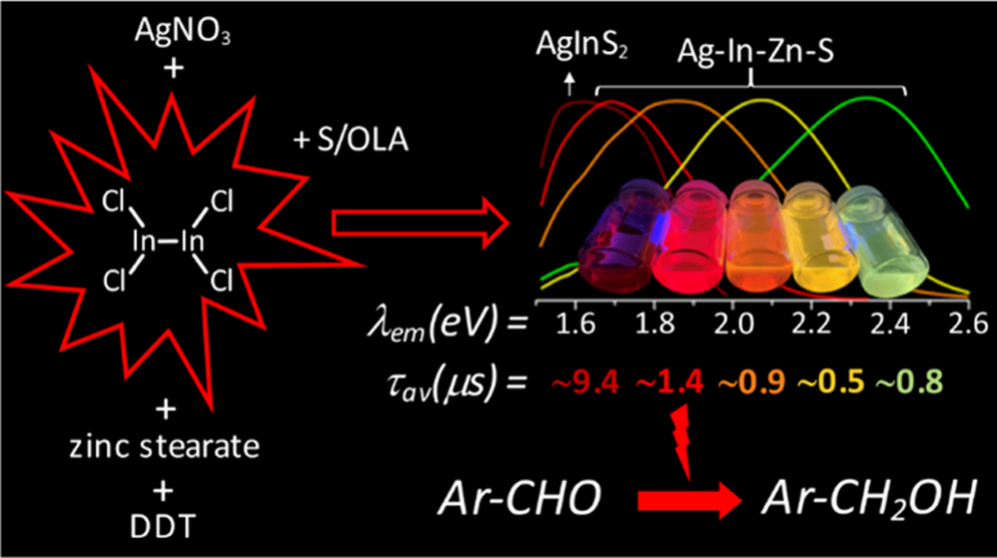

A new indium precursor, namely, indium(II) chloride, was tested as a precursor in the synthesis of ternary Ag–In–S and quaternary Ag–In–Zn–S nanocrystals. This new precursor, being in fact a dimer of Cl_2_In–InCl_2_ chemical structure, is significantly more reactive than InCl_3_, typically used in the preparation of these types of nanocrystals. This was evidenced by carrying out comparative syntheses under the same reaction conditions using these two indium precursors in combination with the same silver (AgNO_3_) and zinc (zinc stearate) precursors. In particular, the use of indium(II) chloride in combination with low concentrations of the zinc precursor yielded spherical-shaped (*D* = 3.7–6.2 nm) Ag–In–Zn–S nanocrystals, whereas for higher concentrations of this precursor, rodlike nanoparticles (*L* = 9–10 nm) were obtained. In all cases, the resulting nanocrystals were enriched in indium (In/Ag = 1.5–10.3). Enhanced indium precursor conversion and formation of anisotropic, longitudinal nanoparticles were closely related to the presence of thiocarboxylic acid type of ligands in the reaction mixture. These ligands were generated *in situ* and subsequently bound to surfacial In(III) cations in the growing nanocrystals. The use of the new precursor of enhanced reactivity facilitated precise tuning of the photoluminescence color of the resulting nanocrystals in the spectral range from *ca*. 730 to 530 nm with photoluminescence quantum yield (PLQY) varying from 20 to 40%. The fabricated Ag–In–S and Ag–In–Zn–S nanocrystals exhibited the longest, reported to date, photoluminescence lifetimes of ∼9.4 and ∼1.4 μs, respectively. It was also demonstrated for the first time that ternary (Ag–In–S) and quaternary (Ag–In–Zn–S) nanocrystals could be applied as efficient photocatalysts, active under visible light (green) illumination, in the reaction of aldehydes reduction to alcohols.

## Introduction

The preparation of highly luminescent semiconductor nanocrystals, which do not contain toxic metals, is of great importance, especially in view of their application in medicine and biomedical sciences.^1−3^ This necessity of eliminating toxic elements resulted in quick development of new preparation methods focused on cadmium- and lead-free nanocrystals of various binary, ternary and quaternary semiconductors.^4,5^ Luminescent binary such as InP^6^ and ternary AgInS_2_ and CuInS_2_^7−10^ nanocrystals are especially interesting in this respect. Colloidal ternary nanocrystals of core/shell structure AgInS_2_/ZnS as well as alloyed quaternary ones (AgInS_2_–ZnS) are highly luminescent, yielding emission tunable in a wide spectral range. For this reason, they are tested as components of quantum dot light-emitting diodes (LEDs)^11−14^ as well as in various types of biomedical applications.^15−19^ Moreover, in recent years, increasing number of reports can be found on their photovoltaic^20−22^ and photocatalytic^23,24^ applications.

In the synthesis of these indium-containing nanocrystals, indium(III) chloride and indium(III) acetate are more frequently used as indium precursors.^25−27^ There also exist reports on the application of indium(III) nitrate, indium(III) acetylacetonate, indium(III) mercaptoacetate, and more recently, InBr_3_ and InI_3_ as precursors.^28,29^ The reason for testing this rather large number of different simple precursors has its origin in their varying reactivities, inherently associated, among others, with their different solubilities in the reaction medium. The second factor of significant importance is the capability of transforming the initial precursor into its active form through binding ligands to cations originating from this precursor. The decomposition of this active form, strongly dependent on the energy of particular bonds breaking and forming new ones, results in the nucleation of nanocrystals.^28,30^ A combination of the above-mentioned factors may lead to products of different chemical compositions.^28,31^

Simple indium precursors are usually used in association with higher fatty acids ligands in varying ratios.^32^ Alternatively salts of indium(III) with higher carboxylic acids can be used, playing a dual role of a metal precursor and a source of ligands.^33^ According to the theory of hard and soft acids and bases, In^3+^ being a hard acid readily binds to hard bases like higher carboxylic acids or amines containing long alkyl substituents.^34^ It should, however, be noted that In^3+^ ions present in the reaction mixture at different stages of nucleation or crystal growth are significantly harder acids than surfacial In^3+^ ions.^35^ Different hard acid character of surfacial In^3+^ ions is clearly manifested by strong effects of ligands on the luminescent properties of indium-containing nanocrystals.^36,37^

**Scheme 1:**
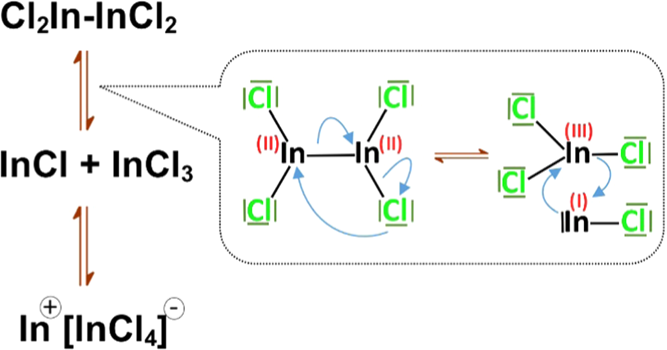
Mechanism of Indium(II) Chloride Disproportionation

There exist several compounds in which the formal oxidation state of indium is lower than +3.^38^ Among them, indium(II) chloride seems especially interesting as a candidate for indium precursor in the synthesis of nanocrystals since it is stable in air and commercially available. In reality, this compound is present in a form of a dimer In_2_Cl_4_. In–In bond in this compound is weak compared to In–Cl one. This leads to its disproportionation with simultaneous formation of In^+^[InCl_4_]^−^ (see Scheme 1).^39^ This disproportionation reaction can be strictly controlled^40,41^ and applied for *in situ* generation of In^3+^ ions in the reaction mixture used in the preparation of nanocrystals. The interest in new precursors containing indium in the oxidation state lower than +3 can be exemplified by a recent report of the use of complexes generated from In(I)Cl in the synthesis of colloidal InAs nanocrystals.^42^

In this paper, we demonstrate for the first time that indium(II) chloride can be used as an efficient precursor in the preparation of highly luminescent inorganic semiconductor nanocrystals varying in composition, shape, and size. In particular, using this precursor it is possible to prepare ternary AgInS_2_ and alloyed quaternary AgInS_2_–ZnS nanocrystals exhibiting strong photoluminescence covering the whole visible range of the spectrum. The new precursor turned out to be more reactive than In(III) precursors investigated to date. Its enhanced reactivity had a pronounced effect on the chemical constitution and morphology of the resulting nanocrystals and on surfacial ligand transformations, previously never observed for this type of nanocrystals.

## Results and Discussion

### Synthesis and Characterization of the Nanocrystals

Similarly to indium(III) chloride, indium(II) chloride is stable under ambient conditions; thus, all operations involving this reagent can be performed in air. A comparison of the reactivities of both types of precursors was made possible by replacing indium(III) chloride with indium(II) chloride in the reaction mixture, while keeping all other reaction conditions unchanged.^43−45^ Ternary Ag–In–S nanocrystals were obtained from a reaction mixture consisting of AgNO_3_ and InCl_2_ precursors, 1-dodecanethiol (DDT), and 1-octadecene (ODE) as a solvent (batch **AIS**). In the case of the synthesis of quaternary Ag–In–Zn–S nanocrystals, zinc stearate was additionally added (batches **A-(1–4)**). An increasing number of **A-X** samples corresponds to increasing zinc content. Before describing the procedure of nanocrystals preparation, it is instructive to discuss the color changes of the reaction mixtures containing InCl_2_ upon their heating from room temperature to 150 °C. At 90 °C, their color changed to yellow, upon further increase of the temperature to 150 °C this color again changed to dark brown. The observed changes were inherently associated with the transformations of InCl_2_ precursor since heating of the precursor mixtures containing InCl_3_ did not cause any visual color change but only gradual dissolution of precursors which yielded colorless, clear liquids. Figure S1 in the Supporting Information presents photographs of both types of reaction mixtures taken at different temperatures. Moreover, in Figure S2 in the Supporting Information, UV–vis spectra, registered for the reaction mixture at different stages of its heating before and after the injection of the sulfur precursor, are compared. This is completed by a short comment explaining these changes.

The reactions leading to nanocrystals formation were initiated by injection of sulfur dissolved in oleylamine (S/OLA) to these mixtures at 150 °C. This was followed by heating to 180 °C. At this temperature, the reaction mixtures were kept for an additional 1 h. Mixtures with InCl_3_ precursors yielded black, insoluble precipitates at the end of the reaction, whereas in the case of the use of InCl_2_ precursors no precipitates could be observed.^45^ Nanocrystals of **AIS** and **A-(1–4)** were separated from the reaction mixture by precipitation with acetone, followed by centrifugation. They were then redispersed in typical nonpolar or weakly polar solvents such as toluene, chloroform, and methylene chloride.

**AIS** nanocrystals were prepared using the metal precursors ratio InCl_2_/AgNO_3_ of 3.0. The application of a significant excess of the precursor of indium was based on experimentally established findings indicating that the presence of defects in nonstoichiometric ternary nanocrystals (In/Ag > 1.0) is beneficial for the resulting photoluminescence quantum yield (PLQY), in accordance with donor–acceptor mechanism of radiative recombination.^45−47^ As expected **AIS** nanocrystals were strongly nonstoichiometric and enriched in indium: **Ag**_**1.0**_**In**_**1.4**_**S**_**2.5**_**(S**_**2.6**_**)**, where the value given in parentheses denotes theoretical content of sulfide anions corresponding to the sum of silver and indium cations. Compositions of nanocrystals of all prepared batches (**AIS** and **A-(1–4)**) are collected in Table 1, whereas their energy-dispersive spectra (EDS) are presented in Figures S3 and S4 in the Supporting Information.

**Table 1:** Precursor Molar Ratios (Silver Nitrate/Indium(II) Chloride/Zinc Stearate/DDT/Sulfur in 1 mL of OLA) and Characteristics of the Synthesized Ternary Ag–In–S and Quaternary Ag–In–Zn–S Nanocrystals: Compositions; Size: Diameter/Width (*D*), Length (*L*), and Aspect Ratio (*L*/*D*)

	Ag/In/Zn/S_DDT_/S_S_	Ag/In/Zn/S(S)	C (wt %)	O (wt %)	Cl (wt %)	S (wt %)	*D* (nm)	*L* (nm)	*L*/*D*
**AIS**	1.0/3.0/–/5.0/2.5	1.0/1.4/–/2.5(2.6)	6.9	0.6	1.1	20.9	9.8 ± 2.3		1.2 ± 0.2
**A**-**1**	1.0/3.0/1.0/5.0/2.5	1.0/1.5/0.3/3.3(3.0)	47.9	8.6	0.6	9.4	6.2 ± 1.1		1.2 ± 0.2
**A-2**	1.0/3.0/3.0/5.0/2.5	1.0/1.5/1.9/3.6(4.6)	45.8	7.3	1.3	9.6	3.7 ± 0.7		1.1 ± 0.1
**A-3**	1.0/3.0/6.0/5.0/2.5	1.0/1.5/4.4/6.8(7.1)	50.3	9.7	1.2	9.5	3.9 ± 0.9	9.0 ± 1.7	2.4 ± 0.7
**A-4**	1.0/3.0/9.0/5.0/2.5	1.0/10.3/12.4/11.8(28.3)	62.7	14.1	0.8	2.8	3.1 ± 0.7	9.9 ± 2.2	3.3 ± 0.9

**Figure 1:**
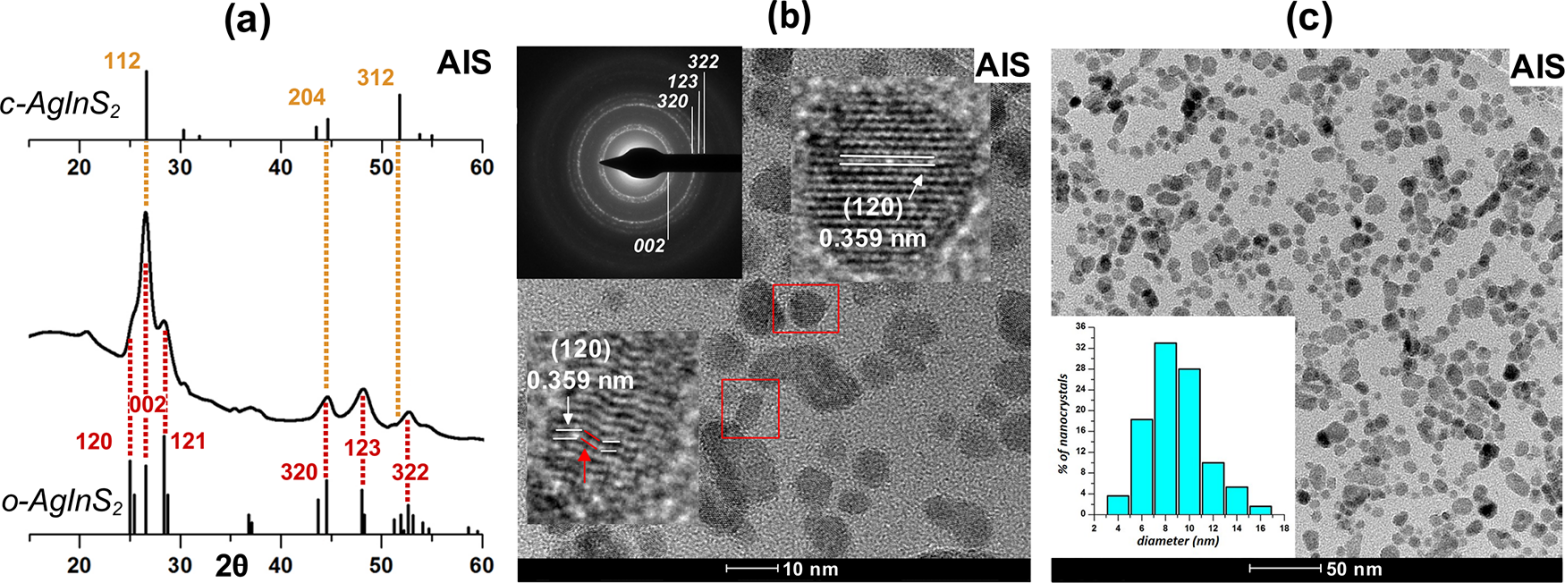
(a) X-ray diffraction (XRD) patterns of **Ag**_**1.0**_**In**_**1.4**_**S**_**2.5**_**(S**_**2.6**_**)** (**AIS**) nanocrystals; (b) HR-TEM image and selected area electron diffraction (SAED) patterns of **Ag**_**1.0**_**In**_**1.4**_**S**_**2.5**_**(S**_**2.6**_**)** (**AIS**); and (c) TEM image and the corresponding histogram of **Ag**_**1.0**_**In**_**1.4**_**S**_**2.5**_**(S**_**2.6**_**)** (**AIS**) nanocrystals.

The powder diffractogram of **AIS** is shown in Figure 1a. The following peaks can be distinguished which can be ascribed to (120), (002), (121), (320), (123), and (322) of orthorhombic AgInS_2_ (JCPDS 00-025-1328). Enhanced intensity of (002) reflection has its origin in the strong overlap of this peak with (121) reflection of chalcopyrite AgInS_2_ (JCPDS 00-25-1330). Orthorhombic-chalcopyrite polytypism was reported for Ag–In–S nanocrystals^48,49^ similarly to wurtzite-chalcopyrite polytypism in the case of Cu–In–S nanocrystals.^50^ The aspect ratio of **AIS** nanocrystals is rather low (1.2 ± 0.20); thus, they can be considered as almost spherical of 9.8 ± 2.3 nm diameter (*n* = 300). Their high-resolution transmission electron microscopy (HR-TEM) and transmission electron microscopy (TEM) images are presented in Figure 1b,c, indicating an interplanar distance of 0.359 nm, which corresponds to the (120) reflection of orthorhombic AgInS_2_.^51−53^ A close inspection of these HR-TEM images reveals the presence of nanocrystals of different orientations of planes, thus corroborating polytypism of the obtained nanoparticles.^50^

In the preparation of quaternary Ag–In–Zn–S nanocrystals (batches **A-(1–4)**), the same composition of the reaction mixture was maintained with a fixed molar ratio of indium to silver precursors (InCl_2_/AgNO_3_ = 3). The only parameter being changed was the content of zinc precursor (zinc stearate) which increased from **A-1** to **A-4**. The most striking difference in chemical compositions of **AIS** nanocrystals and **A-(1–4)** ones was the ratio of inorganic core elements to organic ones. In the case of **AIS** nanocrystals, the elements of the inorganic core predominated with the content of carbon originating from organic ligands being as low as 7 wt %. EDS analysis of **A-(1–4)** nanocrystals yielded carbon contents in the range of 46–63 wt %, clearly showing higher ligand content. This was expected, taking into account a significantly higher surface-to-volume ratio originating from the higher surface-to-volume ratio of **A-(1–4)** nanocrystals compared to the **AIS** ones. For **A-(1–3)** nanocrystals, introduction of the precursor of zinc had no or little effect on the conversion of indium precursor since the determined indium-to-silver ratios (In/Ag = 1.5) were very close to the value found for **AIS** nanocrystals (In/Ag = 1.4). Only for the highest zinc precursor content in the reaction mixture (batch **A-4**), the indium precursor conversion abruptly increased, yielding In/Ag = 10.3 in the resulting nanocrystals.

**Figure 2:**
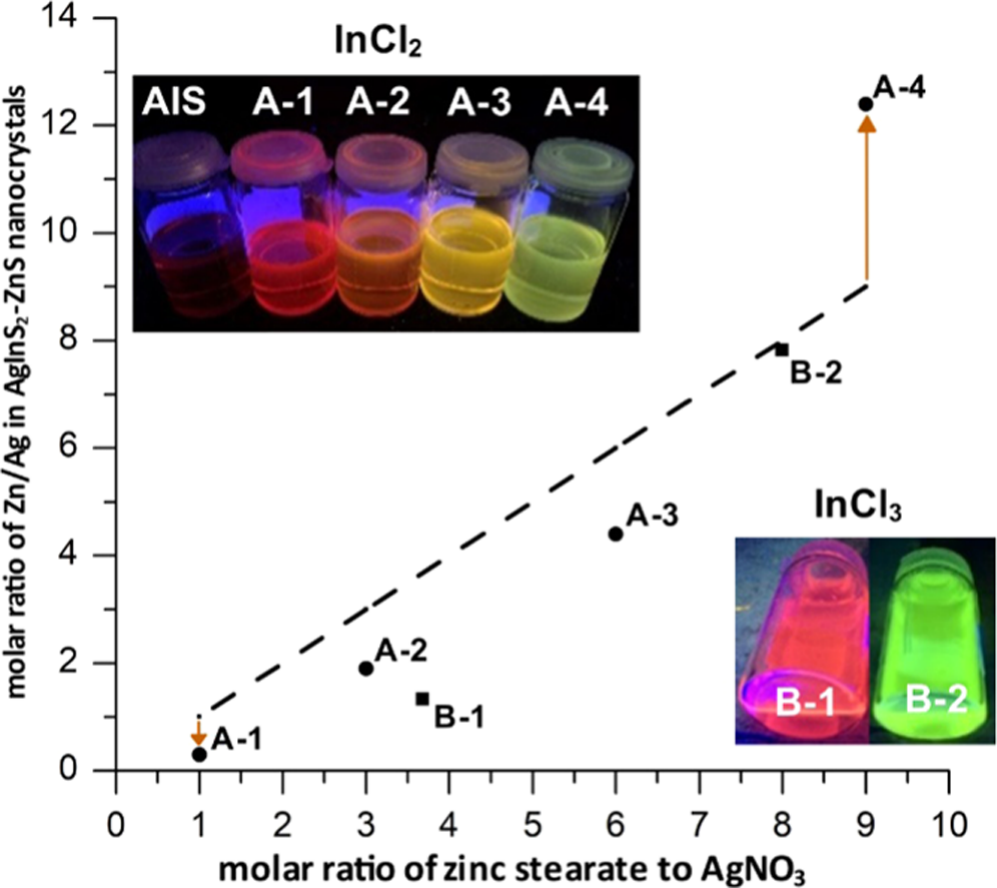
Molar ratio of zinc stearate to AgNO_3_ in the reaction mixture *vs* Zn/Ag ratio in the resulting AgInS_2_–ZnS nanocrystals (molar ratio of indium precursor to AgNO_3_ = 3.0). Circles: this research (InCl_2_) **A-(1–4)**, squares: experimental data (InCl_3_) **B-1** and **B-2** from ref (54). The insets show photographs of UV-illuminated (365 nm) nanocrystals dispersed in toluene solutions.

In Figure 2, Zn/Ag molar ratios in **A-(1–4)** nanocrystals are plotted against the corresponding molar ratios in the reaction mixture. Nanocrystals of **A-(1–3)** batches were enriched in Ag, *i.e*., their Zn/Ag ratios were lower than the molar ratios of their precursors. On the contrary, **A-4** nanocrystals were enriched in Zn since precursors in the molar ratio Zn/Ag = 9.0 yielded nanocrystals of Zn/Ag = 12.4. Additional points in the plot **B-1** (**Ag**_**1.0**_**In**_**2.8**_**Zn**_**1.3**_**S**_**4.0**_**(S**_**6.0**_**)**) and **B-2** (**Ag**_**1.0**_**In**_**1.5**_**Zn**_**7.8**_**S**_**17.0**_**(S**_**10.5**_**)**) represent nanocrystals obtained with InCl_3_ as a precursor, which were reported in ref (54). Elemental analyses of nanocrystals obtained with two different indium precursors (InCl_2_*vs* InCl_3_) indicated distinct differences in their reactivities in the reaction medium. In the case of InCl_2_, with increasing concentration of the precursor of zinc, the conversion of indium precursor remained at a similar level **A-(1–3)**. It abruptly increased for a high degree of zinc precursor conversion (**A-4**). On the contrary, conversion of InCl_3_ dropped with increasing conversion of the precursor of zinc.

**Figure 3:**
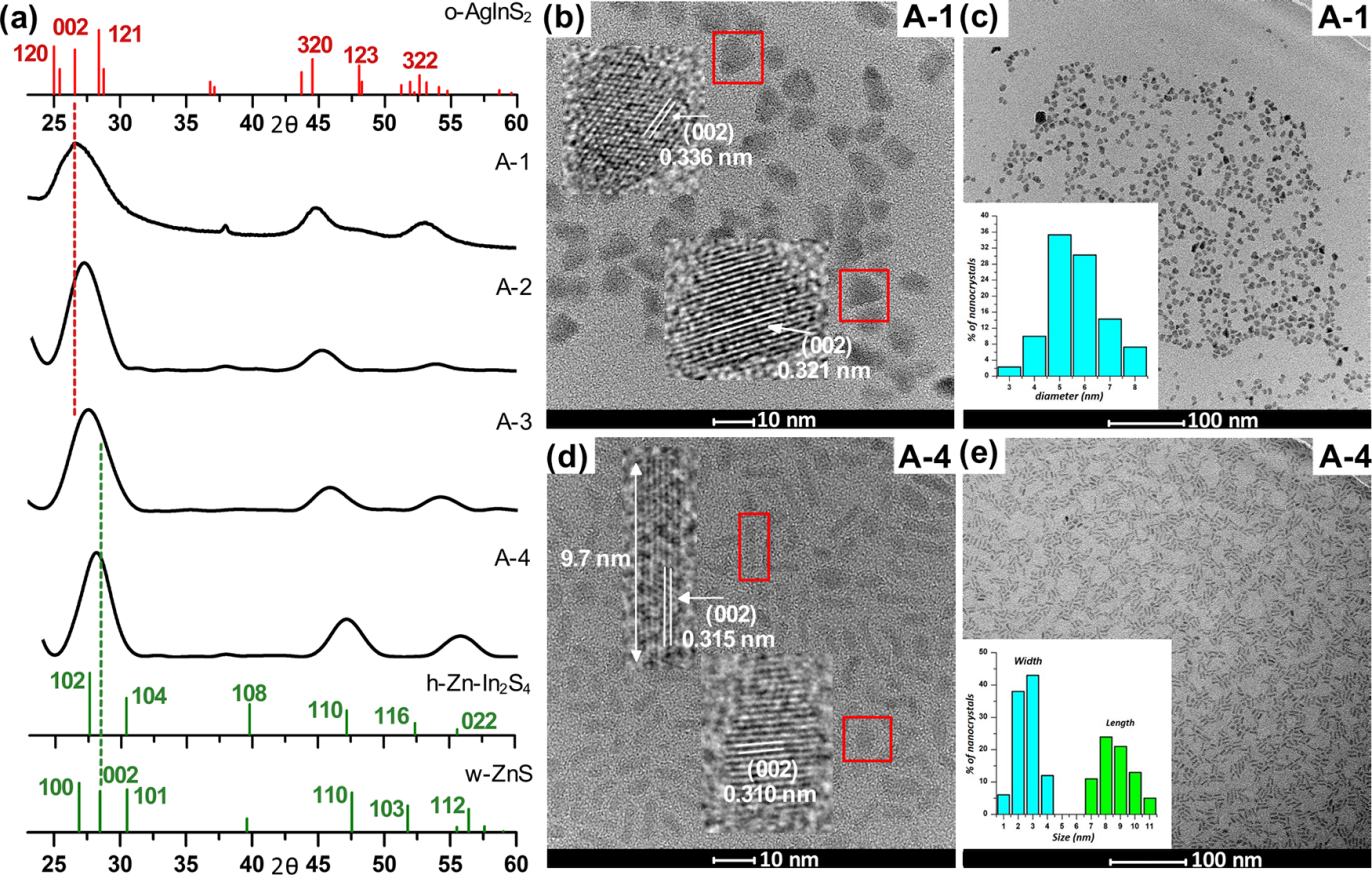
X-ray powder diffractograms of **Ag**_**1.0**_**In**_**1.5**_**Zn**_**0.3**_**S**_**3.3**_**(S**_**3.0**_**)** (**A-1**), **Ag**_**1.0**_**In**_**1.5**_**Zn**_**1.9**_**S**_**3.6**_**(S**_**4.6**_**)** (**A-2**), **Ag**_**1.0**_**In**_**1.5**_**Zn**_**4.4**_**S**_**6.8**_**(S**_**7.1**_**)** (**A-3**), and **Ag**_**1.0**_**In**_**10.3**_**Zn**_**12.4**_**S**_**11.8**_**(S**_**28.3**_**)** (**A-4**) nanocrystals (a), HR-TEM (b, d), and TEM (c, e) images of **Ag**_**1.0**_**In**_**1.5**_**Zn**_**0.3**_**S**_**3.3**_**(S**_**3.0**_**)** (**A-1**) and **Ag**_**1.0**_**In**_**10.3**_**Zn**_**12.4**_**S**_**11.8**_**(S**_**28.3**_**)** (**A-4**) alloyed nanocrystals and their corresponding histograms.

In Figure 3a, powder diffractograms of **A-(1–4)** nanocrystals are presented. Three broadened peaks can be distinguished in each case, whose positions are intermediate between the reflections characteristic of orthorhombic AgInS_2_ (JCPDS 00-025-1328) and hexagonal ZnS (JCPDS 00-036-1450). The effect of zinc content on the position of Bragg reflections could be conveniently followed by analyzing the shift of (002) reflection, which is located at 2θ = 26.6 and 28.5° for orthorhombic AgInS_2_ and hexagonal ZnS, respectively. With increasing zinc content in the studied quaternary nanocrystal (002) reflection was being increasingly shifted toward higher 2θ values: 26.6° (**A-1**), 26.8° (**A-2**), 27.2° (**A-3**), and 28.0° (**A-4**). The introduction of zinc to yield quaternary nanocrystals resulted in a significant decrease of their size from 9.8 ± 2.3 nm in ternary **Ag**_**1.0**_**In**_**1.4**_**S**_**2.5**_**(S**_**2.6**_**)** (**AIS**), to 6.2 ± 1.1 nm in quaternary **Ag**_**1.0**_**In**_**1.5**_**Zn**_**0.3**_**S**_**3.3**_**(S**_**3.0**_**)** (**A-1**). Both **AIS** and **A-1** nanocrystals could be considered as close to spherical but somehow irregular in shape (aspect ratio = 1.2) (see Figures 1 and 3). **A-2** (**Ag**_**1.0**_**In**_**1.5**_**Zn**_**1.9**_**S**_**3.6**_**(S**_**4.6**_**)**) nanocrystals containing more zinc were even smaller (*D* = 3.7 ± 0.7 nm) and were characterized by an aspect ratio reduced to 1.1. Further increase in the content of zinc resulted in a radical change in the shape of nanocrystals from spherical to longitudinal. In the case of **A-3** (**Ag**_**1.0**_**In**_**1.5**_**Zn**_**4.4**_**S**_**6.8**_**(S**_**7.1**_**)**) nanocrystals, their width (3.9 ± 0.9 nm) was comparable to the diameter of **A-2** nanocrystals, but their length was 9.0 ± 1.7 nm, yielding an aspect ratio of 2.4. An even higher aspect ratio (3.3) was obtained for **A-4** (**Ag**_**1.0**_**In**_**10.3**_**Zn**_**12.4**_**S**_**11.8**_**(S**_**28.3**_**)**) nanocrystals characterized by smaller width (3.1 ± 0.7 nm) and higher length (9.9 ± 2.2 nm).

It is instructive to compare HR-TEM images of **A-1** and **A-4** nanocrystals significantly differing in their size, shape, and zinc content (see Figure 3b,d). The interplanar distances derived from these images were in the range of 0.310–0.339 nm and could be ascribed to (002) interplanar distances of orthorhombic AgInS_2_ and hexagonal ZnS, corroborating alloyed-type structure of the studied nanocrystals.^23,45,55,56^ Possible, alternative alloyed-type structures of tetragonal AgInS_2_ (JCPDF 00-25-1330) and cubic ZnS (JCPDF 00-50-0566) can be excluded since in this case significantly shorter interplanar distances, in the range 0.1–0.2 nm should have been observed.^57−59^ Moreover, HR-TEM studies excluded the possibility of the formation of hexagonal InAgZn_2_S_4_-type structures (JCPDF 00-025-0383) since they are characterized by interplanar distances of 0.25–0.29 nm.^60−62^ TEM images of **A-1** and **A-4** nanocrystals are shown in Figure 3c,e. The corresponding images of **A-2** and **A-3** nanocrystals can be found in the Supporting Information.

Nanocrystals **A-1** and **A-4** were compared with the corresponding nanocrystals obtained in the same conditions but using a different indium precursor, *i.e*., InCl_3_. TEM images of these nanocrystals denoted as **B-1** and **B-2** are shown in Figure S7 in the Supporting Information. For small concentrations of the precursor of zinc, no significant differences could be noticed between InCl_3_ and InCl_2_ precursors. In both cases, relatively large, spherical nanocrystals were obtained of very similar size: **A-1** (*D* = 6.2 ± 1.1 nm) and **B-1** (*D* = 6.2 ± 0.9 nm). Large concentrations of zinc precursor in the reaction mixtures resulted in pronounced differentiation in shape and size of nanocrystals obtained using different indium precursors. As already mentioned, in the case of InCl_2_ precursor, longitudinal nanoparticles were formed (**A-4**, *L* = 9.9 ± 2.2 nm; *D* = 3.3 ± 0.9 nm). The use of InCl_3_ precursor resulted in the formation of spherical nanocrystals of smaller size than **A-1** nanoparticles (**B-2**, *D* = 4.2 ± 0.6 nm).

These morphological and composition differences clearly seen in quaternary Ag–In–Zn–S nanocrystals obtained with InCl_2_ and InCl_3_ have their origin in differences in these precursors reactivities and are consistent with our previous findings concerning the reactivity of silver precursors.^63^ If highly reactive silver precursor (AgNO_3_) in combination with InCl_2_ and zinc precursor of low concentration were used, AgInS_2_ phase was formed at the nucleation stage, which was then converted into quaternary Ag–In–Zn–S phase at subsequent stages of the crystal growth. In the case of reaction mixtures of high zinc precursor concentrations, with the highly reactive indium precursor (InCl_2_) concentration unchanged (case of **A-4** nanocrystals), nucleation of ZnIn_2_S_4_ phase took place, which was then followed by the formation of quaternary Ag–In–Zn–S nanocrystals of longitudinal shape. This change in the nanocrystals morphology could be ascribed to the high reactivity of InCl_2_ combined with the high concentration of zinc precursor, both factors favoring anisotropic growth of nanoparticles.^64^ If InCl_3_, exhibiting significantly lower reactivity compared to InCl_2_, was present in the reaction mixture, it preferentially reacted with AgNO_3_ to nucleate the AgInS_2_ phase. This process occurred even in reaction mixtures of high zinc precursor concentrations (case of **B-2** nanocrystals). Nucleation of this type resulted in a quick drop of the precursor concentration and was followed by subsequent growth, converting ternary germs into quaternary Ag–In–Zn–S nanocrystals of spherical shape and relatively low diameter. It should be, however, noted that nucleation *via* ZnIn_2_S_4_ phase formation was previously observed by us even in the presence of the InCl_3_ precursor, but only in the case when a highly reactive silver precursor was not present in the reaction mixture at the stage of nucleation. This was demonstrated by performing a two-step injection of precursors in which the sulfur precursor was injected in the first step followed by the addition of the silver precursor in the second step.^65^

### Optical Properties

Tetragonal AgInS_2_ of chalcopyrite structure is thermodynamically stable at temperatures inferior to 620 °C. Its band gap is equal to 1.87 eV^49,66^ and its exciton Bohr radius is relatively small (5.5 nm).^67^ Orthorhombic AgInS_2_ of pseudowurtzite structure is thermodynamically stable at relatively higher temperatures, its band gap is larger—1.98 eV.^49,66^ The quantum confinement effect is observed for its nanocrystals of diameter inferior to 5 nm,^68^ indicating a similar value of the exciton Bohr radius as in the case of chalcopyrite-type AgInS_2_. The observed increase of the orthorhombic AgInS_2_ band gap can originate from its nonstoichiometry and, in particular, an increased ratio of In/Ag > 1.0. It is postulated that in this case, the upper edge of the valence band corresponds to hybridized S 3p and Ag 4d orbitals, whereas the lower edge of the conduction band is attributed to hybridized S 3p and Ag 4d orbitals.^69^

**Figure 4:**
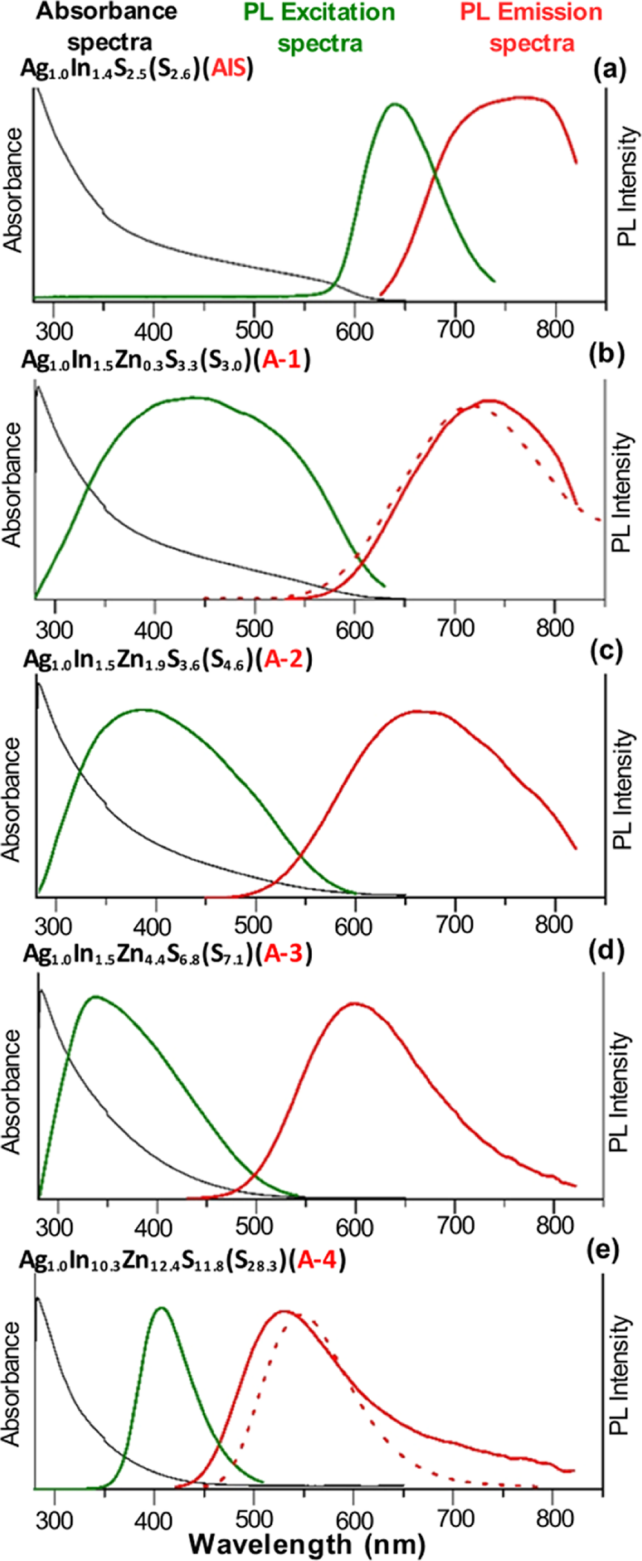
Absorbance, photoluminescence excitation, and emission spectra of toluene dispersion of **Ag**_**1.0**_**In**_**1.4**_**S**_**2.5**_**(S**_**2.6**_**)** (**AIS**) (a) and alloyed **Ag**_**1.0**_**In**_**1.5**_**Zn**_**0.3**_**S**_**3.3**_**(S**_**3.0**_**)** (**A-1**) (b), **Ag**_**1.0**_**In**_**1.5**_**Zn**_**1.9**_**S**_**3.6**_**(S**_**4.6**_**)** (**A-2**) (c), **Ag**_**1.0**_**In**_**1.5**_**Zn**_**4.4**_**S**_**6.8**_**(S**_**7.1**_**)** (**A-3**) (d), and **Ag**_**1.0**_**In**_**10.3**_**Zn**_**12.4**_**S**_**11.8**_**(S**_**28.3**_**)** (**A-4**) (e) nanocrystals. For comparison, the emission spectra (dot lines) of toluene dispersions of **Ag**_**1.0**_**In**_**2.8**_**Zn**_**1.3**_**S**_**4.0**_**(S**_**6.0**_**)** (**B-1**) and **Ag**_**1.0**_**In**_**1.5**_**Zn**_**7.8**_**S**_**17.0**_**(S**_**10.5**_**)** (**B-2**) alloyed nanocrystals are presented.

UV–vis–near-infrared (NIR) of ternary (**AIS**) and quaternary (**A-(1–4)**) nanocrystals dispersed in toluene are presented in Figure 4. On the basis of these spectra, optical band gaps were determined following the relationship (*Ah*ν)^2^*vsh*ν (see Figure S8 in the Supporting Information). *E*_g(opt)_ ∼ 2.0 eV was determined for **AIS** (**Ag**_**1.0**_**In**_**1.4**_**S**_**2.5**_**(S**_**2.6**_**)**), nanocrystals, a value very close to that of bulk orthorhombic AgInS_2_. This was not unexpected since no quantum confinement was expected for these relatively large nanoparticles (9.8 ± 2.3 nm). Essentially the same *E*_g(opt)_ ∼ 2.0 eV was calculated for **A-1** (**Ag**_**1.0**_**In**_**1.5**_**Zn**_**0.3**_**S**_**3.3**_**(S**_**3.0**_**)**) nanocrystals characterized by small content of zinc and relatively large size (6.2 ± 1.1 nm). *E*_g(opt)_ steadily enlarged with increasing content of zinc: 2.4 eV (**A-2**), 2.7 eV (**A-3**) and 3.2 eV (**A-4**). These values consistently fell in the range determined by the bands of orthorhombic AgInS_2_ (1.98 eV)^66^ and hexagonal ZnS (3.68 eV).^70^

Photoluminescence of binary nanocrystals such as CdSe is governed by a simple mechanism of radiative recombination (1S_(e)_ → 1S_3/2(h)_) between the states located in the vicinity of the band edge. This gives rise to a very narrow emission peak (full width at half-maximum, fwhm, of 80–150 meV at room temperature), small Stokes shift (21 ± 4 meV), and room-temperature radiative decay times of the order of 18–40 ns. Spectral parameters are also dependent on the size of nanocrystals (quantum confinement) and the presence of surfacial defects, which may quench the photoluminescence.^71−73^ In ternary nanocrystals such as AgInS_2_ as well as in core/shell AgInS_2_/ZnS, alloyed AgInS^2^–ZnS and nonstoichiometric Ag–In–Zn–S (In/Ag ≠ 1.0) ones, the emission is governed by the donor–acceptor pair recombination mechanism.^74^ The donor states are located in the band gap, below the lower edge of the conduction band and are associated with the presence of S vacancies and interstitial Ag atoms. The acceptor states located above the upper edge of the valence band originate from the presence of Ag vacancies and interstitial S atoms.

**Figure 5:**
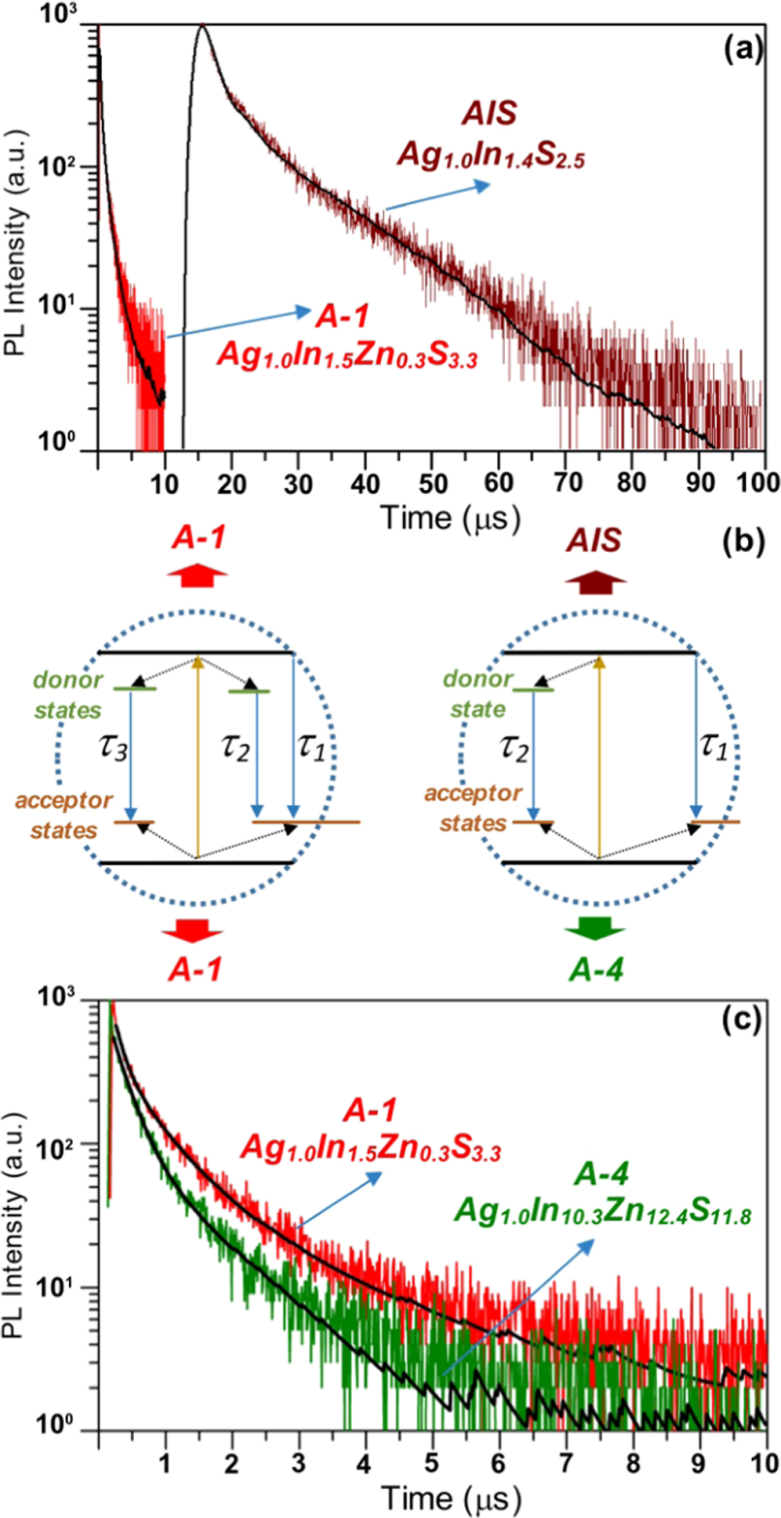
Photoluminescence decay curves of **Ag**_**1.0**_**In**_**1.4**_**S**_**2.5**_**(S**_**2.6**_**)** (**AIS**) (a) and alloyed **Ag**_**1.0**_**In**_**1.5**_**Zn**_**0.3**_**S**_**3.3**_**(S**_**3.0**_**)** (**A-1**) (a, c) and **Ag**_**1.0**_**In**_**10.3**_**Zn**_**12.4**_**S**_**11.8**_**(S**_**28.3**_**)** (**A-4**) (c) nanocrystals and the corresponding bi (**AIS** and **A4**) and three (**A-1**)-exponential fitting curves. Schematic of the relaxation dynamics proposed for **AIS**, **A-1**, and **A-4** nanocrystals (b).

Surfacial defects in the forms of vacancies, dangling bonds, and oxygen adatoms may also contribute to this mechanism.^68,75^ Radiative recombination mechanism in ternary AgInS_2_ nanocrystals and related alloyed and nonstoichiometric nanoparticles gives rise to broad emission peaks (fwhm ∼ 0.4 eV) and large Stokes shifts.^74^ These characteristic spectral features are rationalized by strong electron–phonon interactions of trapped carriers.^68^ Deeper elucidation of these phenomena requires the use of time-resolved emission spectroscopy techniques. Decay curves registered for ternary AgInS_2_ and core/shell AgInS_2_/ZnS ones are of complex character and cannot be fitted with a single-exponential function and usually require the application of two^23,68,74,76,77^ or three-exponential models.^17,24,49,75,78,79^ The two-exponential model yields two decay times: the longer one (τ_2_) is usually ascribed to the photoluminescence originating from the bulk of the nanocrystal according to the above-described donor–acceptor pair mechanism. The shorter decay time (τ_1_) is related to the photoluminescence involving surfacial defects.^68^ In the case of photoluminescence of ternary AgInS_2_ and CuInS_2_ nanocrystals which had to be fitted using three-exponential model, different interpretations could be found in the literature explaining the presence of the third exponent.^75,80,81^ For example, detailed analysis of the photoluminescence decay in alloyed AgInS_2_–ZnS nanocrystals clearly demonstrated the necessity of fitting the obtained data using three-exponential functions. The shortest decay time (τ_1_) was attributed to the presence of surficial defects, the intermediate (τ_2_) and the longest (τ_3_) decay times could be tentatively ascribed to the recombination between the donor and acceptor states at the surface and in the bulk of a nanocrystal, respectively.^75^ These briefly outlined photoluminescence mechanisms are schematically illustrated in Figure 5b. It should, however, be noted that such characteristic Stokes-shifted and long-lived luminescence was also observed for stoichiometric AgInS_2_ and CuInS_2_ nanocrystals. This was rationalized by theoretical and experimental investigations as effects originating from the detailed structure of the valence band featuring two sublevels with different parity.^82,83^

UV–vis–NIR absorption spectra as well as excitation and stationary photoluminescence spectra of **AIS** and **A-(1–4)** nanocrystals are compared in Figure 4. Photoluminescence decay curves registered for **AIS**, **A-1**, and **A-4** nanocrystals are presented in Figure 5a,c. The corresponding curves obtained for **A-2** and **A-3** nanocrystals are shown in Figure S9 in the Supporting Information. The decay profiles were fitted using two (**AIS** and **A-4**) and three (**A-1**, **A-2**, and **A-3**) exponential models yielding high accuracy, as indicated by the narrow distribution of fitting residuals (see eqs 1 and 2)^84^
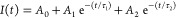
1
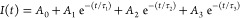
2where *I*(*t*) is the photoluminescence intensity (au); τ_1_, τ_2_, and τ_3_ are the photoluminescence lifetime components (ns); *A*_0_ is the background noise level; and *A*_1_, *A*_2_, and *A*_3_ represent the relative weight of the decay components (%) at *t* = 0.

For all fitting functions, the obtained χ^2^ values were in the range of 0.98–1.1, proving adequacy of the applied fitting method. Average photoluminescence lifetimes were calculated according to eq 3
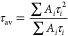
3where τ_av_ denotes the average photoluminescence decay time (ns) and *A*_*i*_ and τ_*i*_ are the normalized amplitude (%) and the lifetime (ns) of the (*i*) component, respectively.

All optical parameters determined for ternary (**AIS**) and quaternary (**A**-**(1–4)**) nanocrystals are collected in Table 2.

**Table 2:** Optical Band Gaps, Maxima of the Photoluminescence Bands (PL), Quantum Yields (QY), Tri- and Biexponential Fit Parameters, χ^2^ Values, and Average Emission Lifetimes (τ_av_) for Ternary Ag–In–S and Quaternary Ag–In–Zn–S Nanocrystals

sample	*E*_g(opt)_ (eV)	PL (nm)	QY (%)	τ_1_ (ns)	*A*_1_ (%)	τ_2_ (ns)	*A*_2_ (%)	τ_3_ (ns)	*A*_3_ (%)	χ^2^	τ_av_ (ns)
**AIS**	2.0	755	12.0	1151	83.4	13 120	16.6			1.10	9458
**A-1**	2.0	731	40.0	139	16.5	591	50.7	1921	32.8	1.07	1460
**A-2**	2.4	664	21.0	2	0.9	343	46.9	1081	52.1	0.98	917
**A-3**	2.7	596	33.3	84	13.3	331.5	56.5	1051	30.2	1.09	500
**A-4**	3.2	528	20.0	251	63.6	1039	36.4			1.06	805

The excitation spectrum of **AIS** (**Ag**_**1.0**_**In**_**1.4**_**S**_**2.5**_**(S**_**2.6**_**)**) nanocrystals consisted of a relatively narrow peak with a clear maximum at 625 nm located close to the absorption threshold in the UV–vis–NIR spectrum and roughly corresponding to the optical band gap (∼2.0 eV). Its emission peak was broad showing a maximum at ∼755 nm. The introduction of even small amounts of zinc to yield quaternary **A-1** (**Ag**_**1.0**_**In**_**1.5**_**Zn**_**0.3**_**S**_**3.3**_**(S**_**3.0**_**)**) nanocrystals resulted in profound spectroscopic changes. In particular, the excitation peak broadened covering the spectral range of 300–600 nm. Its maximum was hypsochromically shifted to ∼450 nm. In addition, a significant increase of PLQY was observed from 12% in the case of **AIS** nanocrystals to 40% for **A-1** ones. **A-1** nanocrystals emitted red light (λ_max_ = 731 nm). With growing zinc content the emission peak was being increasingly hypsochromically shifted to λ_max_ = 664 nm for **A-2** (**Ag**_**1.0**_**In**_**1.5**_**Zn**_**1.9**_**S**_**3.6**_**(S**_**4.6**_**)**) nanocrystals and λ_max_ = 596 nm for **A-3** (**Ag**_**1.0**_**In**_**1.5**_**Zn**_**4.4**_**S**_**6.8**_**(S**_**7.1**_**)**) nanocrystals. **A-4** (**Ag**_**1.0**_**In**_**10.3**_**Zn**_**12.4**_**S**_**11.8**_**(S**_**28.3**_**)**) nanocrystals which were the richest in zinc emitted green light (λ_max_ = 528 nm). It should be pointed out that its excitation peak was relatively narrow, contrary to the cases of **A-1**, **A-2**, and **A-3** nanocrystals. Moreover, its maximum at *ca*. 400 nm closely corresponded to the absorption threshold in the UV–vis–NIR spectrum as well as to the optical band gap of 3.1–3.2 eV. It should be noted that these relatively narrow excitation spectra of **AIS** and **A-4** are not typical. However, several examples of excitation spectra of similar shapes were reported for a variety of nanocrystals, for example for CuInS_2_–ZnS^85^ and AgInS_2_–ZnS nanocrystals^69,86^ The excitation spectrum of AgInS_2_–ZnS presented in ref (87) was essentially identical in shape to the spectra of **AIS** and **A-4**.

The spectroscopic peculiarity of **AIS** (**Ag**_**1.0**_**In**_**1.4**_**S**_**2.5**_) nanocrystals was manifested in its decay curves fitted by two-exponential functions (Figure 5a). This fitting resulted in extremely long decay times τ_1_ and τ_2_ of ∼1.1 and ∼13.1 μs leading to an average value (τ_av_) of 9.4 μs—the highest ever reported for ternary Ag–In–S nanocrystals. Typical decay times reported for this class of nanocrystals were in the range of 3–4 μs.^88^ Decay curves of quaternary nanocrystals had to be fitted with three-exponential functions. The results of these fittings showed that τ_av_ values determined for quaternary nanocrystals (**A-(1–3)**) were 1 order of magnitude smaller than that calculated for ternary (**AIS**) ones. Moreover, they were dependent on zinc content decreasing from 1.5 to 0.5 μs when going from **A-1** to **A-3**. τ_1_ values were also strongly reduced to 2–140 ns. Finally, τ_2_ and τ_3_, *i.e*., decay times reflecting donor–acceptor radiative processes associated with the nanocrystal core and surface, respectively, were of the order of 0.3 and 1.0 μs. It should also be noted that for quaternary nanocrystals, the contribution of surficial processes characterized by τ_1_ was significantly smaller than in the case of **AIS** nanocrystals, dropping to 1% for **A-2** nanocrystals, where the donor–acceptor radiative recombination mechanism predominated.

The richest in zinc **A-4** (**Ag**_**1.0**_**In**_**10.3**_**Zn**_**12.4**_**S**_**11.8**_**(S**_**28.3**_**)**) nanocrystals behaved distinctly different. Its decay curve could be fitted with two-exponential functions (see Figure 5b,c) yielding τ_av_ ∼ 0.8 μs. The calculated τ_1_ and τ_2_ were 251 and 1039 ns, respectively. Compared to **A-(1–3)** nanocrystals, the contribution of surficial mechanisms characterized by τ_1_ was significantly more pronounced (>60%), whereas the contribution of donor–acceptor recombination occurring in the core diminished to *ca*. 36% (see Table 2). These distinct differences between decay curves of **A-(1–3)** and **A-4** and the different nature of its excitation spectrum are perfectly in line with our already expressed conclusion suggesting different nucleation mechanisms of these zinc-rich nanocrystals which involves the formation of ZnIn_2_S_4_ rather than AgInS_2_ as in the case of **A-(1–3)** nanocrystals.

Comparison of nanocrystals prepared using the InCl_2_ precursor with those synthesized applying InCl_3_ in identical conditions, *i.e*., **B-1** in the same conditions as **A-1** and **B-2** in the same conditions as **A-4**, clearly showed that the dissimilarity of their spectroscopic properties originated from significant differences in their chemical compositions caused by the use of indium precursors of different reactivities. **B-1** (**Ag**_**1.0**_**In**_**2.8**_**Zn**_**1.3**_**S**_**4.0**_**(S**_**6.0**_**)**) and **B-2** (**Ag**_**1.0**_**In**_**1.5**_**Zn**_**7.8**_**S**_**17.0**_**(S**_**10.5**_**)**) emitted red (720 nm) and green (543 nm) light, respectively, consistent with their different Zn/Ag ratios.^54^ It should be noted that **A-4** and **B-2** exhibit similar emission spectra with maxima at 528 and 542 nm, respectively, despite different shapes and sizes. In the case of ternary or quaternary nanocrystals of sizes inferior to their exciton Bohr radii, the luminescence band position is a complex product of their composition and the quantum confinement phenomenon. The hypsochromic shift of the photoluminescence band of **A-4** to the green region of the visible spectrum is most probably caused by the high content of Zn in these quaternary nanocrystals, although the quantum confinement effect can also interfere, taking into account their rather small diameter (*L* = 9.9 ± 2.2 nm, *D* = 3.3 ± 0.9 nm). The same two factors influence the position of the photoluminescence band of spherical **B-2** nanocrystals, the effect of quantum confinement being probably more pronounced (*D* = 3.3 ± 0.9 nm). It is difficult to separate these two factors but their combination may lead to similar spectra of nanocrystals differing in composition, shape, and size. The application of more reactive indium(II) chloride significantly extended the range of nanocrystals compositions possible to obtain. For example, Zn/Ag ratios could be varied from 0.3 to 12.4, allowing for precise tuning of the photoluminescence in the spectral range from 755 to 528 nm.

To summarize this part of the research, the use of InCl_2_ as a new precursor of indium in the preparation of semiconductors nanocrystals resulted in the preparation of ternary Ag–In–S and quaternary Ag–In–Zn–S nanoparticles which distinctly differed in their morphological and spectroscopic properties from nanocrystals synthesized using different precursors of indium and stabilized either with hydrophobic or hydrophilic ligands. Detailed comparison of spectroscopic properties of nanocrystals prepared in this research and other ternary and quaternary nanocrystals of this family, reported in the literature, is presented in Table S1 in the Supporting Information.

In this respect, special attention should be paid to extremely long decay times of τ_av_ ∼ 9.4 and ∼1.5 μs, determined for **AIS** (**Ag**_**1.0**_**In**_**1.4**_**S**_**2.5**_**(S**_**2.6**_**)**) and **A-1** (**Ag**_**1.0**_**In**_**1.5**_**Zn**_**0.3**_**S**_**3.3**_**(S**_**3.0**_**)**) nanocrystals, respectively. Further investigations of these nanocrystals should be carried out in view of their promising applications in bioimaging, photocatalysis, and photovoltaics.^68,88^

### Surface Characterization

**Figure 6:**
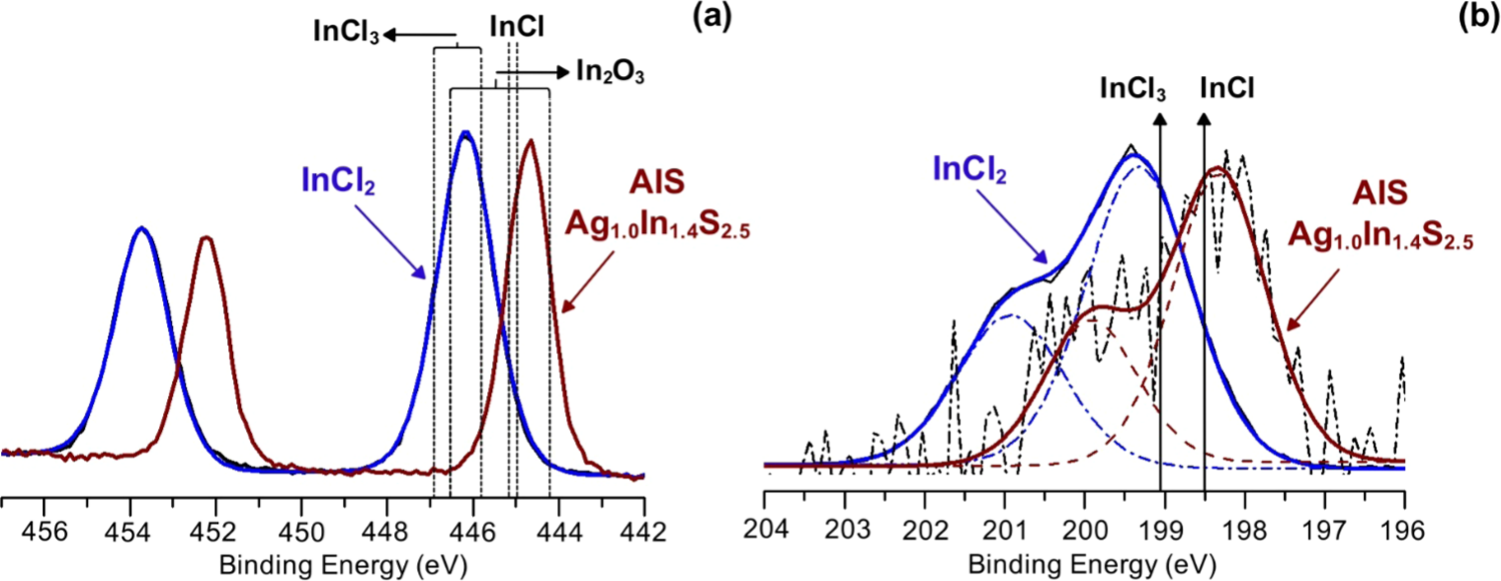
In 3d (a) and Cl 2p (b) high-resolution XPS spectra in the surface region of indium(II) chloride and **Ag**_**1.0**_**In**_**1.4**_**S**_**2.5**_**(S**_**2.6**_**)** (**AIS**) nanocrystals. Binding energy of the reference substances: InCl_3_, InCl, and In_2_O_3_ are also shown.

InCl_2_ precursor as well as the nanocrystals prepared with its use (**AIS**, **A-1**, and **A-4**) were additionally studied by X-ray photoelectron spectroscopy (XPS). The resulting survey spectra are presented in Figures S10 and S11 in the Supporting Information. In Figure 6a,b, high-resolution XPS (HR-XPS) spectra of indium(II) chloride are presented. In the XPS In 3d spectrum, only one doublet is present at 446.2 eV (In 3d_5/2_) and 453.7 eV (In 3d_3/2_). The measured binding energy *E*_b_, is distinctly different than that of In(I) (444.9–445.1 eV),^89,90^ but it falls in the range reported for InCl_3_ (445.9–446.9 eV)^89,90^ and In_2_O_3_ (444.3–446.7 eV).^91,92^ Similarly, *E*_b_ derived from the XPS Cl 2p spectrum (199.3 eV for Cl 2p_3/2_) is close to that reported for InCl_3_ (199.1 eV) but is clearly different from the value reported for InCl (198.5 eV).^90^ The presence of only one type of indium and chlorine seems to favor the dimeric form of the precursor (Cl_2_In–InCl_2_), characterized by strong contribution of the In–In covalent bond, rather than its disproportionated form of In(I)In(III)Cl_4_ where two oxidation states of indium are expected. As seen from the survey spectrum, the content of carbon and oxygen were of the order 0.05 wt %. These impurities were identified as water and organic origin contaminants by recording HR-XPS C 1s and O 1s (see Figure S12 in the Supporting Information).

In Figure 6a, XPS In 3d spectrum of **AIS** (**Ag**_**1.0**_**In**_**1.4**_**S**_**2.5**_**(S**_**2.6**_**)**) nanocrystals is shown. Similar to that in the case of InCl_2_, only one doublet can be observed, excluding nonequivalence of indium surfacial atoms. It should be noted that *E*_b_ values 444.7 eV (In 3d_5/2_) and 452.2 eV (In 3d_3/2_) are lower than the corresponding values in InCl_2_, indicating oxidation of In(II) to In(III). Indeed, *E*_b_ measured for In 3d_5/2_ is in the range reported of ternary Ag–In–S and quaternary Ag–In–Zn–S of different compositions and prepared using In(III)-based precursors (443.5–445.0 eV).^62,93−95^ Similar shift by *ca*. 1 eV toward lower *E*_b_ values is observed for residual chlorine (*ca*. 1 wt %) in **AIS** (**Ag**_**1.0**_**In**_**1.4**_**S**_**2.5**_**(S**_**2.6**_**)**) nanocrystals (Figure 6b). The determined values are: 198.3 eV (Cl 2p_3/2_) and 199.9 eV (Cl 2p_1/2_). Lower *E*_b_ values of In 3d_5/2_ and Cl 2p_3/2_ measured for these nanocrystals compared to InCl_3_ and InCl seem to indicate the effect of stoichiometric differentiation of the surface, originating predominantly from the presence of ligands, which significantly lower the hard acid character of In(III). A similar phenomenon was reported for InP nanocrystals.^35^

In Figure S13 in the Supporting Information, Ag 3d, In 3d, and Zn 2p HR-XPS registered for ternary **AIS** (**Ag**_**1.0**_**In**_**1.4**_**S**_**2.5**_**(S**_**2.6**_**)**) as well as quaternary **A-1** (**Ag**_**1.0**_**In**_**1.5**_**Zn**_**0.3**_**S**_**3.3**_**(S**_**3.0**_**)**) and **A-4** (**Ag**_**1.0**_**In**_**10.3**_**Zn**_**12.4**_**S**_**11.8**_**(S**_**28.3**_**)**) nanocrystals. No significant differences can be seen in the measured *E*_b_ values: Ag 3d_5/2_ ∼367 eV, In 3d_5/2_ ∼444.6 eV, and Zn 2p_3/2_ ∼1021 eV. They all fall in the range reported for ternary Ag–In–S and quaternary Ag–In–Zn–S nanocrystals.^62,93−95^

**Figure 7:**
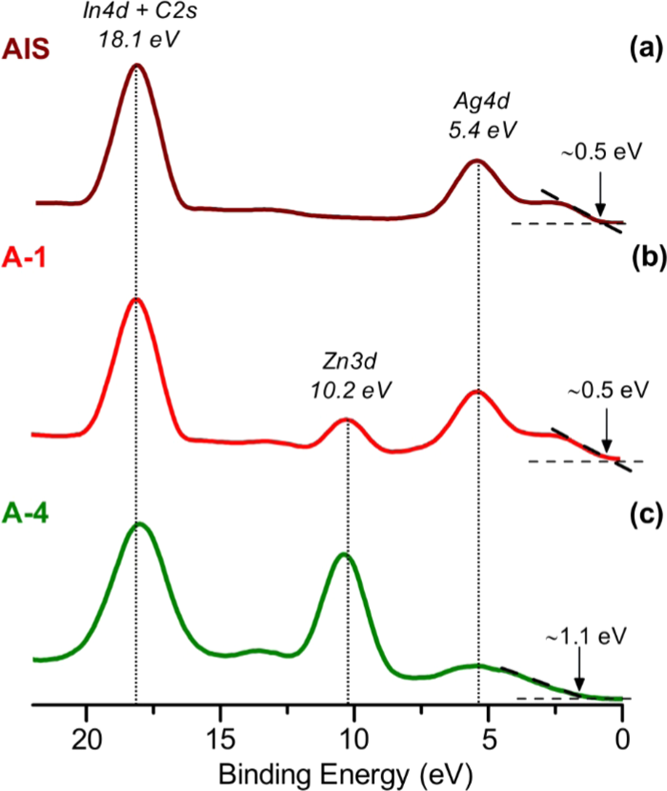
Comparison of the valence bands XPS spectra of **Ag**_**1.0**_**In**_**1.4**_**S**_**2.5**_**(S**_**2.6**_**)** (**AIS**), **Ag**_**1.0**_**In**_**1.5**_**Zn**_**0.3**_**S**_**3.3**_**(S**_**3.0**_**)** (**A-1**), and **Ag**_**1.0**_**In**_**10.3**_**Zn**_**12.4**_**S**_**11.8**_**(S**_**28.3**_**)** (**A-4**) nanocrystals.

Figure 7 shows the valence band region of the XPS spectra of **AIS**, **A-1**, and **A-4** nanocrystals. In all three samples, signals at ∼18.1 and ∼5.4 eV can be seen, originating from In 4d + C 2s and Ag 4d orbitals. For nanocrystals containing zinc (**A-1** and **A-4**), an additional peak can be noticed at 10.2 eV, ascribed to the Zn 3d orbital.^69,96,97^ It should be noted that the onset of the least energetic peak in the spectra of **AIS** and **A-1** is located near ∼0.5 eV whereas in the case of **A-4** it is shifted to ∼1.1 eV. This finding is consistent with the differences in their optical band gaps derived from UV–vis–NIR investigation (see Figure S8 in the Supporting Information). **AIS** and **A-1** show similar band gaps (*E*_g_ ∼ 2.0 eV), whereas the band gap of **A-4** is significantly larger (*E*_g_ ∼ 3.2 eV).

S 2p, C 1s, and O 1s HR-XPS studies are especially useful in analyzing binding of ligands to the nanocrystal surface. HR-XPS S 2p spectra of **AIS** (**Ag**_**1.0**_**In**_**1.4**_**S**_**2.5**_**(S**_**2.6**_**)**), **A-1** (**Ag**_**1.0**_**In**_**1.5**_**Zn**_**0.3**_**S**_**3.3**_**(S**_**3.0**_**)**) and **A-4** (**Ag**_**1.0**_**In**_**10.3**_**Zn**_**12.4**_**S**_**11.8**_**(S**_**28.3**_**)**) nanocrystals are presented in Figure S14a–c, Supporting Information. Spectra of **AIS** and **A-1** can be deconvoluted to two and three doublets, respectively, indicating certain nonequivalence of surficial sulfur atoms. The dominant doublet at ∼161 eV (S 2p_3/2_) can be ascribed to the structural (S^2–^) form of sulfur. The second doublet of weak intensity at 162.9 eV (S 2p_3/2_) and 162.6 eV (S 2p_3/2_) in the spectra of **AIS** and **A-1**, respectively, should be ascribed to surficial sulfur forming a covalent bond with carbon atoms of the ligand (C–S). This type of covalent bonding may be formed during chemical processes of redox nature occurring in the reaction mixture and accompanying the nanocrystal growth, as demonstrated previously.^44,98^ In the case of **A-1** nanocrystals, an additional doublet can be noticed 166.9 eV (S 2p_3/2_) corresponding to oxidized forms of surficial sulfur (SO*_x_*).^44,99^

HR-XPS C 1s spectra (see Figure S14d–f) provide information concerning surfacial ligands. The studied nanocrystals of different compositions (**AIS**, **A-1**, and **A-4**) exhibit similar spectral features. The dominant signal at 284.6 eV corresponds to aliphatic carbons of long-chain ligands, whereas signals in the range of 285.2–285.6 eV can be ascribed to these carbon atoms of the ligand, which are located in close vicinity of a functional group. Thus, they can also be attributed to carbons covalently bound to surfacial sulfur atom (C–S) which is consistent with the HR-XPS S 2p spectra (*vide supra*). In the case of **AIS** and **A-4** nanocrystals, additional signals can be seen at *ca*. 286 and 288 eV, which originate from the presence of ether (C–O) and carbonyl (C=O) types of carbon.^100^ O 1s HR-XPS spectra are presented in Figure S15 in the Supporting Information. In the case of **AIS** nanocrystals, only one signal corresponding to oxygen in organic ligands can be distinguished in the spectral range of 531.7–531.9 eV. Deconvolution of the spectra of **A-1** and **A-4** gives rise to an additional peak of small intensity at *ca*. 530 eV which can be ascribed to In_2_O_3_.^100^

NMR techniques applied directly to the identification of organic ligands in colloidal dispersions of the nanocrystals provide very limited information due to significant differences in the relaxation times of different groups of nonequivalent protons. This especially applies to protons of anchor groups, directly bound to the nanocrystal surface, for which partial or total quenching of the proton signals may occur.^45,101^ Thus, to gain more information on surfacial ligands, in addition to spectra recorded for dispersions of **AIS**, **A-1**, and **A-4** nanocrystals we registered spectra of organic residue recovered after selective dissolution of the inorganic core. This approach was previously developed in our group and applied to the analysis of nanocrystals of different nature.^101,102^

**Figure 8:**
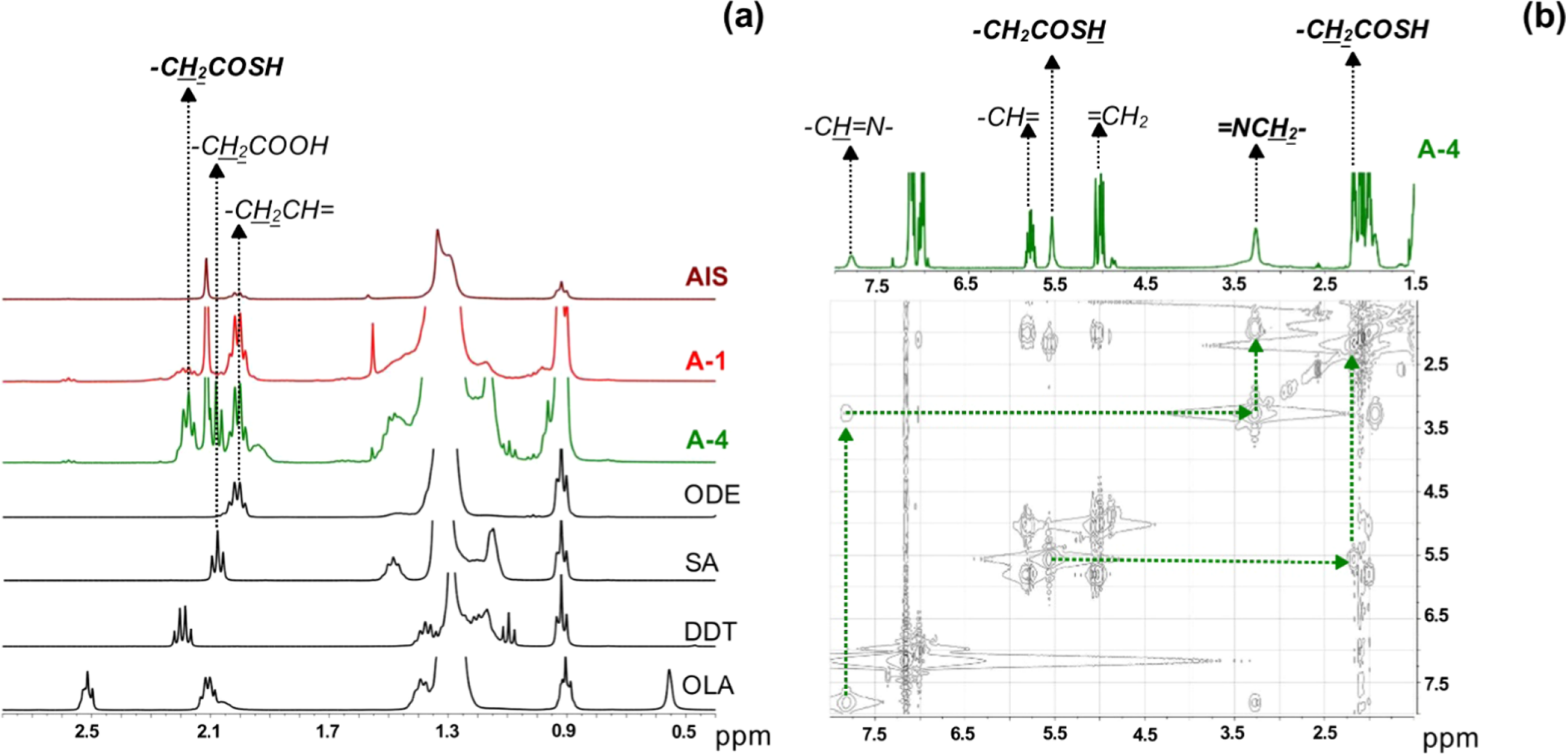
(a) ^1^H NMR spectra of the organic residue from **Ag**_**1.0**_**In**_**1.4**_**S**_**2.5**_**(S**_**2.6**_**)** (**AIS**), **Ag**_**1.0**_**In**_**1.5**_**Zn**_**0.3**_**S**_**3.3**_**(S**_**3.0**_**)** (**A-1**), and **Ag**_**1.0**_**In**_**10.3**_**Zn**_**12.4**_**S**_**11.8**_**(S**_**28.3**_**)** (**A-4**) nanocrystals, 1-octadecene (ODE), stearic acid (SA), 1-dodecanethiol (DDT), and oleylamine (OLA) recorded in C_6_D_6_, (b) ^1^H–^1^H COSY NMR spectrum of the organic residue (in C_6_D_6_) from **Ag**_**1.0**_**In**_**10.3**_**Zn**_**12.4**_**S**_**11.8**_**(S**_**28.3**_**)** (**A-4**) nanocrystals.

**Scheme 2:**
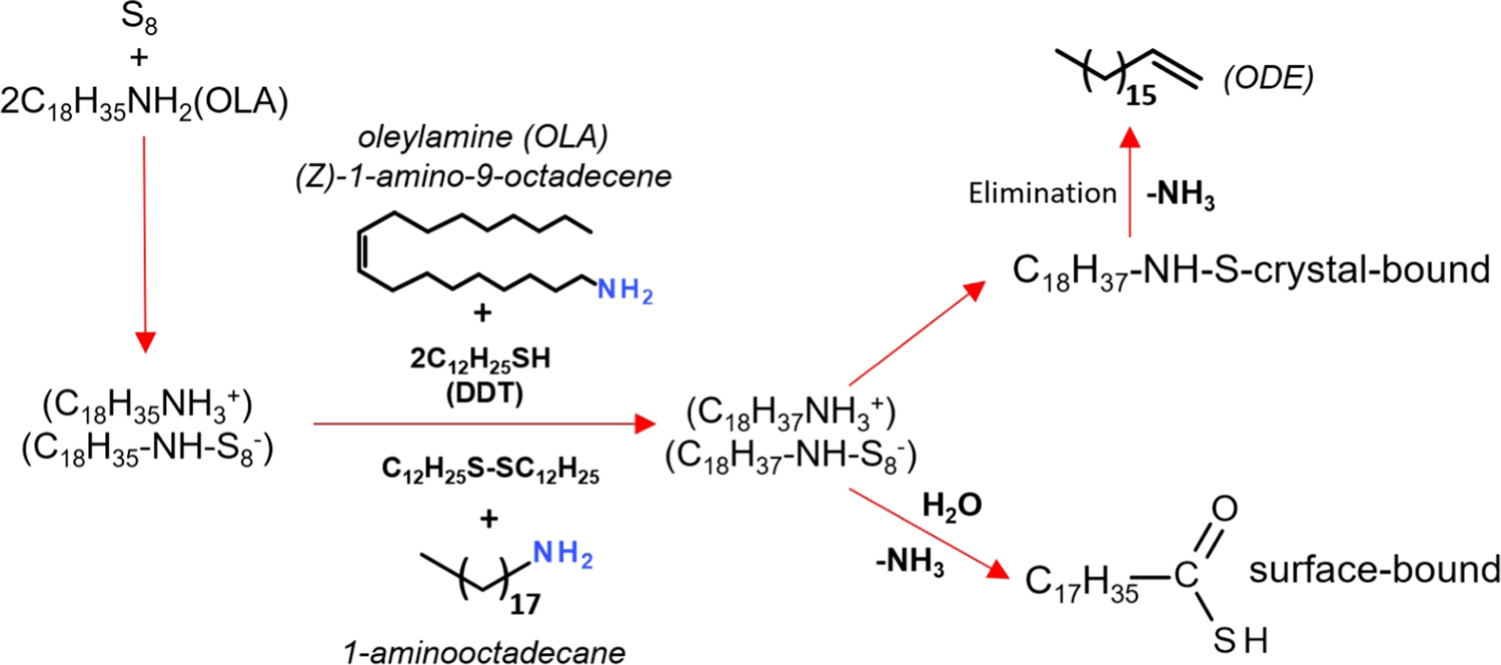
Proposed Reaction Pathways in the Reaction Mixtures Used for the Preparation of Ag–In–S and Ag–In–Zn–S Nanocrystals

In Figure S16 in the Supporting Information, ^1^H NMR spectra recorded for colloidal dispersions of **AIS**, **A-1**, and **A-4** nanocrystals in C_6_D_6_ are compared with the corresponding spectra of their organic residue collected after the inorganic core dissolution. In the case of **AIS** (**Ag**_**1.0**_**In**_**1.4**_**S**_**2.5**_**(S**_**2.6**_**)**) nanocrystals, the ^1^H NMR spectrum of the colloidal dispersion is very similar to that registered for the organic residue. In both spectra, only signals characteristic of a long-chain alkene can be found, namely, multiplets in the following spectral ranges: 1.98–2.04 ppm (−CH_2_–CH=), 4.91–5.02 ppm (=CH_2_), and 5.77–5.85 ppm (−CH=). They undoubtedly originate from the presence of 1-octadecene. Although this compound was used as a solvent, its presence is not associated with incomplete solvent removal. It is formed as a product of the decomposition of nanocrystals stabilizing ligands, according to Scheme 2. This conclusion is supported by the results of our previous studies confirming the presence of ODE in NMR spectra of various nanocrystals such as Ag–In–Zn–S^45^ or Cu_2_ZnSnS_4_^102^ despite the fact that this compound was not present in the initial reaction mixture. ^1^H NMR spectra of colloidal dispersions of **A-1** and **A-4** nanocrystals and their organic residues are distinctly different. In the spectra of the colloidal dispersions, the lines are broadened whereas in the spectra of organic residues clear multiplets can be distinguished, allowing for unequivocal identification of the investigated chemical species. In Figure 8a, ^1^H NMR spectra of recovered organic residues of **AIS**, **A-1**, and **A-4** nanocrystals are presented. In the spectrum of the organic part of **A-4**, in addition to a multiplet at 1.98–2.04 ppm (−CH_2_–CH=CH_2_) originating from the presence of ODE, a triplet appears 2.08 ppm (−CH_2_COOH), which can be ascribed to stearic acid (SA). In the spectral range of 2.16–2.21 ppm, a multiplet is present which can be correlated with the signal at 5.56 ppm (see ^1^H–^1^H correlated spectroscopy (COSY) spectrum in Figure 8b), which strictly excludes the presence of thiol. To a first approximation, coupling between these signals might originate from the following segment −CH_2_–CH=CH–CH_2_– characteristic of OLA. However, in view of the absence of a signal at 2.52 ppm, which is diagnostic of −CH_2_– adjacent to −NH_2_, this possibility has to be excluded. This can be further corroborated by a comparison of the spectrum of colloidal **A-4** nanocrystals with that of its organic residue (see Figure S16e,f in the Supporting Information).

In the spectrum of the colloidal dispersion, a broadened signal at ∼2.2 ppm can be noticed together with another broadened signal at ∼5.62 ppm, the latter being shifted with respect to the corresponding signal in the solution spectrum of the organic residue. This shift can be considered as a spectroscopic manifestation of binding of the ligand to the nanocrystal surface. In the case of OLA bound to the nanocrystal surface, this shift is not observed as the functional group is distant from the binding site. The above short discussion clearly shows that in the course of the nanocrystal synthesis, chemical transformation of ligands may occur, leading to new ligands of different chemical identity. Below, this problem is discussed in detail.

It is known that the dissolution of elemental sulfur in OLA results in a formation of an active precursor of the following chemical formula (C_18_H_35_NH_3_^+^)(C_18_H_35_NH-S_8_^–^), which can consecutively be transformed to carboxylic acids and thioacids.^103,104^ It can therefore be postulated that in the reaction mixture, a thiocarboxylic acid is formed of the formula R-CH_2_(C=O)SH, which gives rise to a multiplet at 2.21 ppm corresponding to the methylene group (−CH_2_−) adjacent to the functional group, whereas the broadened signal appearing at 5.56 ppm has to be ascribed to −SH bound to the nanocrystal surface.^105^ A detailed analysis of the ^1^H–^13^C heteronuclear multiple bond correlation (HMBC) NMR spectrum registered for the organic residue of **A-4** is presented in Figure S17 in the Supporting Information in view of the unequivocal confirmation of the chemical structure of this thioacid-type ligand. In ^1^H NMR spectra of all nanocrystals dispersions (**AIS**, **A-1**, and **A-4**), this broadened signal, originating from R-CH_2_(C=O)SH, is present in the spectral range of 5.00–5.75 ppm (see Figure S16 in the Supporting Information). This can be considered as a proof that the ligand is not a side product of the inorganic core dissolution process but it was formed *in situ* in the reaction mixture. In the case of **AIS** and **A-1** nanocrystals, the content of this thiocarboxylic acid ligand is rather small, in contrast to **A-4** nanocrystals where additional broadened and signals are observed at 3.27 and 7.80 ppm. Strong coupling between these signals suggests the presence of imine-type groupings (−CH_2_–N=CH−).^106^ Their formation can be considered as a proof of hydrogenation of DDT and OLA, yielding 1-aminooctadecane, which binds to the nanocrystal surface (C_18_H_17_NH–S-crystal). In the inorganic core dissolution process, this ligand is eliminated in a form of ODE. Chemical transformations occurring in the reaction during the nanocrystals growth and stabilization are depicted in Scheme 2. The total absence of signals attributable to DDT and OLA can be considered as an indirect proof of the validity of the proposed transformation mechanism.

XPS and NMR results presented here were almost perfectly complementary. XPS analysis of **AIS** (**Ag**_**1.0**_**In**_**1.4**_**S**_**2.5**_**(S**_**2.0**_**)**) clearly indicated the presence of carbonyl groups, despite the fact that compounds containing this group were present in the reaction mixture. Through complementary NMR analysis, it was unequivocally demonstrated that thiocarboxylic acids, *i.e*., compounds comprising carbonyl groups were formed in the reaction medium through transformation of the precursor of sulfur into these chemical entities. Moreover, these analyses indicated a clear correlation between the nanocrystal’s chemical composition (in particular the content of sulfur) and the presence of a given type of ligands. **AIS** (**Ag**_**1.0**_**In**_**1.4**_**S**_**2.5**_**(S**_**2.0**_**)**) and **A-1** (**Ag**_**1.0**_**In**_**1.5**_**Zn**_**0.3**_**S**_**3.3**_**(S**_**3.0**_**)**) nanocrystals, enriched in sulfur as seen from the elemental EDS analysis, could be characterized by an equilibrated surface and were stabilized by crystal-bound C_18_H_17_NH–S-crystal ligands. Upon dissolution of the inorganic core these ligands were eliminated in a form of ODE. NMR spectra of organic residues of these nanocrystals revealed only minute amounts of typical coordinating ligands containing thiocarboxylic anchor groups. In the case of **A-1**, signals ascribed to sulfur of higher oxidation states could also be observed.

Elemental analysis of **A-4** (**Ag**_**1.0**_**In**_**10.3**_**Zn**_**12.4**_**S**_**11.8**_**(S**_**28.3**_**)**) nanocrystals clearly indicated that they were strongly cation-enriched, representing unequilibrated surface. NMR spectra of their organic residues revealed the presence of thiocarboxylates and stearates as ligands bound to indium and zinc, respectively. Although there exist several reports on coordination compounds of indium(III) and thiocarboxylate ligands,^107^ no indium-containing inorganic nanocrystals capped with thiocarboxylates have been reported to date.

In the case of **B-1** (**Ag**_**1.0**_**In**_**2.8**_**Zn**_**1.3**_**S**_**4.0**_**(S**_**6.0**_**)**) and **B-2** (**Ag**_**1.0**_**In**_**1.5**_**Zn**_**7.8**_**S**_**17.0**_**(S**_**10.5**_**)**), *i.e*., nanocrystals prepared under the same experimental conditions as **A-1** (**Ag**_**1.0**_**In**_**1.5**_**Zn**_**0.3**_**S**_**3.3**_**(S**_**3.0**_**)**) and **A-4** (**Ag**_**1.0**_**In**_**10.3**_**Zn**_**12.4**_**S**_**11.8**_**(S**_**28.3**_**)**) nanocrystals, respectively, but using less reactive InCl_3_ precursor instead of the InCl_2_ one, the NMR analysis revealed two different types of stabilizing ligands. These were stearate anions originating from the precursor of zinc and 1-aminooctadecane ligands being the product of OLA hydrogenation and acting as “crystal-bound” or “surface-bound” ligands.^45^

To summarize this part of the paper, application of a more reactive indium precursor, namely, InCl_2_ instead of InCl_3_, results in the formation of nanocrystals capped with thiocarboxylate anions as ligands. It should be noted here that carboxylate ligands have not been detected in nanocrystals obtained from the InCl_3_ precursor under the same experimental conditions. Thiocarboxylates are less hard bases than stearate anions which stabilize nanocrystals obtained with InCl_3_. Surfacial In(III) ions in the resulting Ag–In–Zn–S quaternary nanocrystals constitute an example of less hard acids compared to bulk (macrocrystalline) indium ions. Moreover, the use of this more reactive InCl_2_ precursor allows for significant extension of the obtainable nanocrystals compositions and, by consequence, facilitates precise tuning of the nanocrystals photoluminescence spectra.

### Photocatalytic Activity

In the last few years, several papers appeared devoted to the application of inorganic semiconductors nanocrystals as photocatalyst of many important reactions, mainly the reduction ones^108,109^ and C–C coupling reactions.^110^ In the majority of cases, cadmium chalcogenide nanocrystals, including core/shell ones, were used for this purpose.^111^ The most important drawback of the above-mentioned photocatalysts is their toxicity. However, a few reports of the use of nontoxic nanocrystals, such as core–shell binary InP/ZnS, appeared in the literature.^112^ It is therefore tempting to verify whether alloyed or nonstoichiometric ternary (Ag–In–S) and quaternary (Ag–In–Zn–S) can be used as photocatalysts. Apart from nontoxicity, the great advantage of this family of nanocrystals is the possibility of tuning their optical properties in a wide spectral range.

**Figure 9:**
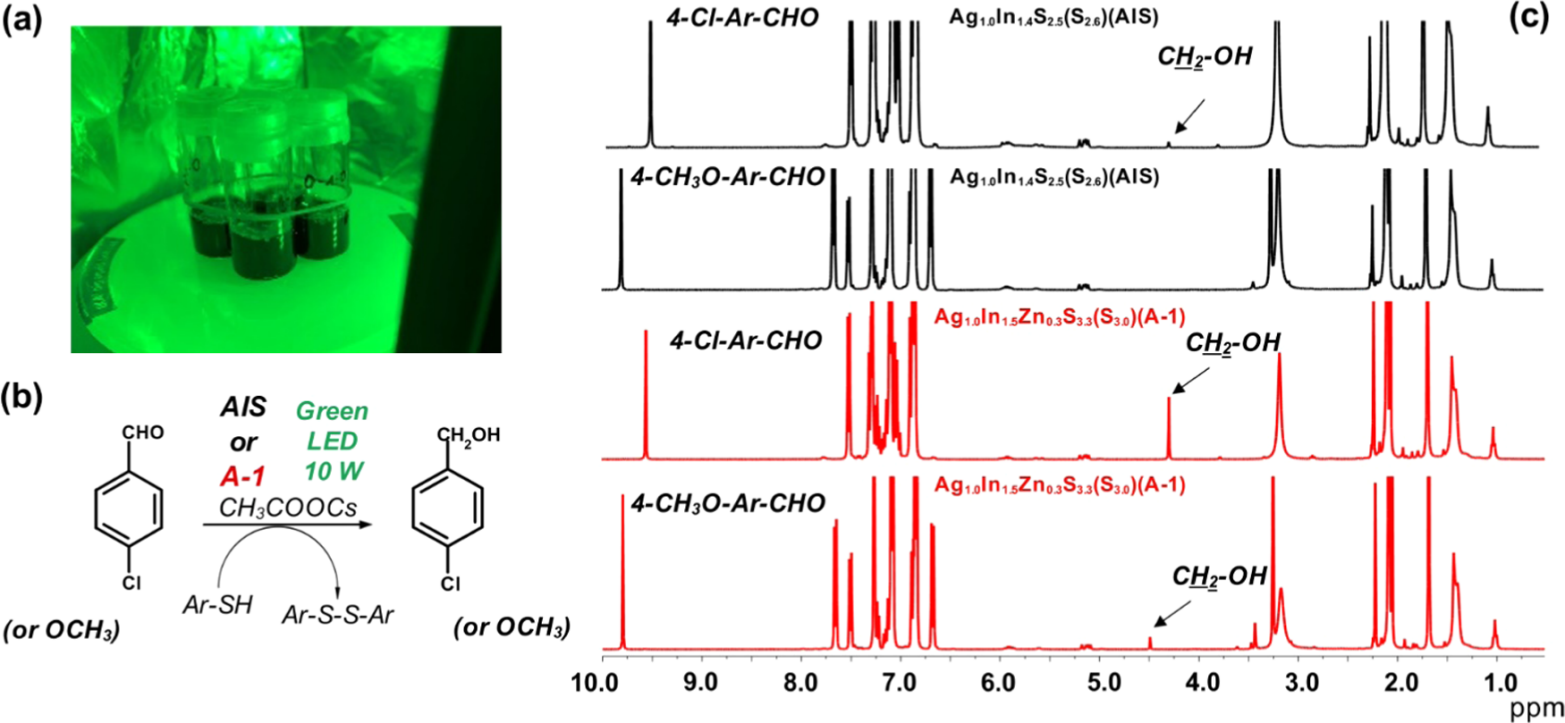
(a) Setup for investigation of photocatalytic reactions under 10 W green LED (λ = 528 nm) irradiation, (b) photocatalytic reduction of aldehydes to alcohols, (c) ^1^H NMR spectra of the photocatalytic reduction reaction mixture used for photocatalytic reduction of 4-chlorobenzaldehyde or 4-methoxybenzaldehyde with **AIS** (**Ag**_**1.0**_**In**_**1.4**_**S**_**2.5**_**(S**_**2.0**_**)**) or **A-1** (**Ag**_**1.0**_**In**_**1.5**_**Zn**_**0.3**_**S**_**3.3**_**(S**_**3.0**_**)**) nanocrystals as photocatalysts (in C_6_D_6_). All four processes were carried out under the same conditions.

In the research described here, **AIS** (**Ag**_**1.0**_**In**_**1.4**_**S**_**2.5**_**(S**_**2.0**_**)**) and **A-1** (**Ag**_**1.0**_**In**_**1.5**_**Zn**_**0.3**_**S**_**3.3**_**(S**_**3.0**_**)**) were tested as photocatalysts in aldehyde reduction reactions, following the procedure elaborated previously for CdSe/CdS core/shell nanocrystalline photocatalysts.^109^ Thiols are frequently used as reducing agents, which readily bind to the nanocrystal surface. Two aldehydes were selected for test reactions, namely, 4-chlorobenzaldehyde and 4-methoxybenzaldehyde. The same reaction set was used in all four cases: CH_3_COOCs/*p*-toluenethiol/nanocrystals (**AIS** or **A-1**) in benzene-*d*_6_. This solvent was purposely used with the goal of monitoring the changes in the reaction mixture by ^1^H NMR. The catalytic tests were carried out at room temperature using an LED monochromatic source (λ = 528 nm) of 10 W power (see Figure 9a,b).

In Figure 9c, ^1^H NMR spectra of reaction mixtures, exposed to green light irradiation, for 5 h, are compared. For 4-chlorobenzaldehyde and 4-methoxybenzaldehyde, the diagnostic −CHO singlets can be distinguished at 9.55 and 9.79 ppm. Lines at 4.28 and 4.49 ppm correspond, in turn, to methylene protons in the −CH_2_OH units of the reaction products. The ratio of the integrated intensities of these lines allows for direct determination of the conversion degree. **AIS** (**Ag**_**1.0**_**In**_**1.4**_**S**_**2.5**_**(S**_**2.0**_**)**) is a rather inefficient photocatalyst, and the conversion of aldehyde to alcohol is ∼1% for 4-chlorobenzaldehyde. No conversion products were detected in the case of 4-methoxybenzaldehyde.

Significantly higher photocatalytic activity was measured for **A-1** (**Ag**_**1.0**_**In**_**1.5**_**Zn**_**0.3**_**S**_**3.3**_**(S**_**3.0**_**)**). In the reaction of 4-chlorobenzaldehyde reduction, the conversion degree reached 50%, whereas in the case of 4-methoxybenzaldehyde, it was equal to 10%. Thus, these preliminary results confirmed the photocatalytic activity of both types (**AIS** and **A-1**) nanocrystals. Moreover, they demonstrated the inactivity of thiols as reducing agents under these experimental conditions. The observed differences in the photocatalytic activity of **AIS** and **A-1** could be rationalized by inspection of their excitation spectra (see Figure 4). The spectrum of **AIS** (**Ag**_**1.0**_**In**_**1.4**_**S**_**2.5**_**(S**_**2.0**_**)**) presents a narrow peak with a clear maximum at 625 nm, whereas a broad band, covering the spectral range from 300 to 600 nm and comprising green radiation, can be noticed in the spectrum of **A-1** (**Ag**_**1.0**_**In**_**1.5**_**Zn**_**0.3**_**S**_**3.3**_**(S**_**3.0**_**)**). Finally, a clear substituent effect on the photocatalytic activity should be noted. The presence of an electron accepting substituent (chlorine) in the para position promoted the photocatalytic reduction. On the contrary, electron-donating methoxy substituent in the same position lowered the photocatalytic activity of **AIS** and **A-1** nanocrystals in the same experimental conditions as those reported for CdSe/CdS photocatalysts.^109^ For comparative reasons, we performed the same catalytic tests using **B-1** nanocrystals as photocatalysts and carried out the experiments under identical conditions. It should be noted that the emission spectrum of **B-1** nanocrystals was very similar to that measured for **A-1**. This could imply similar photocatalytic properties. However, no reduction products were detected in this case **B-1** sample (see Figure S18, Supporting Information), clearly demonstrating catalytic inactivity of these nanocrystals and advantageous properties of nanocrystals prepared from the InCl_2_ precursor. Finally, it should be noted that the presented results of photocatalytic studies should be treated as preliminary. Further investigations are needed leading to the optimization of the catalyst composition and reaction conditions.

## Conclusions

A new indium precursor, namely, indium(II) chloride in its dimeric form (In_2_Cl_4_) turned out to be significantly more reactive in the process of ternary (Ag–In–S) and quaternary (Ag–In–Zn–S) nanocrystals preparation compared to traditionally used indium(III) chloride. For low and moderate concentrations of zinc precursor (zinc stearate), the conversion of the new In_2_Cl_4_ precursor was comparable to that of the traditional InCl_3_ one. For the highest concentrations of zinc stearate, the conversion of the new indium precursor was significantly higher than that of InCl_3_, indicating different nucleation mechanisms. In the case of low zinc stearate concentrations, AgInS_2_ germs were formed as a consequence of high reactivities of silver and indium precursors, the resulting nanocrystal were spherical in shape. Increasing concentration of zinc stearate led to the formation of ZnIn_2_S_4_ germs and longitudinal quaternary Ag–In–Zn–S nanocrystals in the crystal growth step.

The observed changes in the composition, shape, and size of nanocrystals originated from chemical transformations of the reaction mixture leading to ligands of different chemical nature. Ternary Ag–In–S nanocrystals and quaternary Ag–In–Zn–S nanocrystals of low indium and zinc contents were capped with ligands covalently bound to structural sulfur atoms (C_18_H_17_NH–S-crystal), *i.e*., ligands formed *in situ* in the reaction mixture as a result of its chemical transformations. Quaternary Ag–In–Zn–S nanocrystals of high indium and zinc contents were capped with thiocarboxylate and stearate anionic ligands bound to indium and zinc cations, respectively. Again, thiocarboxylate ligands were formed as products of the reaction mixture chemical transformations.

Nanocrystals obtained with the highly reactive In_2_Cl_4_ indium precursor exhibited interesting photoluminescent properties. First, their photoluminescence color could be controllably tuned in the spectra range from 730 to 530 nm, always exhibiting the PLQY values in the range of 20–40%. Second, for ternary Ag–In–S nanocrystals enriched in indium, the longest, ever reported for this family of compounds photoluminescence lifetime of τ_av_ ∼ 9.4 μs, was found. A long photoluminescence lifetime of ∼1.4 μs was also measured for quaternary Ag–In–Zn–S nanocrystals. Finally, quaternary nanocrystals prepared from the InCl_2_ precursor turned out to be promising photocatalysts in the reaction of aldehydes reduction to alcohols.

## Experimental Section

### Materials

Silver nitrate (99%), indium(II) chloride (99%) indium(III) chloride (98%), zinc stearate (technical grade), 1-dodecanethiol (DDT, 98%), sulfur (99%), 1-octadecene (ODE, 90%), oleylamine (OLA, 70%), and benzene-*d*_6_ (100%, 99.6 atom % D) were supplied by Sigma-Aldrich.

### Preparation of the S/OLA Precursor

Sulfur powder (15 mg, 0.47 mmol) and OLA (1.0 mL) were loaded into a glass vial, which was then immersed in an ultrasonic bath. The mixture was sonicated at room temperature (for about 10 min) until a clear red solution was formed.

### Preparation of Ag–In–S and Alloyed Ag–In–Zn–S Nanocrystals

All operations were carried out under constant dry argon flow. Silver nitrate (0.03 g, 0.18 mmol), indium(II) chloride (0.11 g, 0.59 mmol), DDT (0.20 g, 1.0 mmol) and zinc stearate (0.11–1.11 g, 0.18–1.8 mmol) were mixed with ODE (15 mL) in three-neck flask. The mixture was heated to 150 °C until a homogeneous solution was formed. Then, 1 mL of S/OLA precursor was quickly injected into the reaction solution. The temperature was increased to 180 °C, and the mixture was kept at this temperature for 60 min. After the mixture was cooled to room temperature, toluene (10 mL) was added, and the reaction mixture was centrifuged—the isolated black precipitate consisting of organic waste and agglomerated particles was separated (however, usually no solids were isolated in this step). The supernatant was treated with 30 mL of acetone, leading to the precipitation of the desired fraction of nanocrystals. The nanocrystals were separated by centrifugation (7000 rpm, 5 min) and then redispersed in toluene (or hexane, chloroform, dichloromethane).

### Ligand Recovery

A colloidal solution of nanocrystals (in 10 mL of toluene) and 10 mL of concentrated HCl were placed in a screw-capped ampule, which was vigorously shaken for about 60 min. Next, 20 mL of water was added and the as-obtained mixture was centrifuged (15 000 rpm, 5 min) to achieve phase separation. Solids were discarded. The organic phase was collected, and the aqueous phase was extracted with 10 mL chloroform. The combined organic extracts were washed two times with water, evaporated, and dried under reduced pressure.

### Procedures for the Reduction of Aldehyde

To a solution of 4-chlorobenzaldehyde (0.6 mmol, 84 mg) or 4-methoxybenzaldehyde (0.6 mmol, 82 mg), CH_3_COOCs (0.1 mmol, 20 mg), and *p*-toluenethiol (3.0 mmol, 372 mg) in benzene-*d*_6_ (4 mL) were added **Ag_1.0_In_1.4_S_2.5_(S_2.0_)** (**AIS**) (∼10 mg in 1 mL of benzene-d_6_) or **Ag_1.0_In_1.5_Zn_0.3_S_3.3_(S_3.0_)** (**A-1**) (∼10 mg in 1 mL of benzene-*d*_6_) nanocrystals. The vial was sealed and purged with argon for 15 min before illumination by a 10 W green LED (λ = 528 nm).

## Supporting Information Available

Characterization methods, photographs of the reaction mixtures, EDS spectra, TEM and HR-TEM images of Ag–In–S and Ag–In–Zn–S nanocrystals, UV–vis–NIR, photoluminescence and decay curves of Ag–In–Zn–S nanocrystals, XPS survey and HR-XPS spectra of indium precursor and Ag–In–S and Ag–In–Zn–S nanocrystals, ^1^H NMR spectra of C_6_D_6_ dispersion of Ag–In–S and Ag–In–Zn–S nanocrystals capped with initial ligands and ligands recovered after dissolution of nanocrystals, ^1^H–^13^C HMBC NMR spectrum of the organic residue from Ag–In–Zn–S nanocrystals, detailed comparison of spectroscopic properties of nanocrystals prepared in this research and other ternary and quaternary nanocrystals reported in the literature (PDF)
