# Supplementary material for: Indium(II) Chloride as a Precursor in the Synthesis of Ternary (Ag–In–S) and Quaternary (Ag–In–Zn–S) Nanocrystals
Source: Chem Mater. 2022 Jan 3;34(2):809–25. doi: 10.1021/acs.chemmater.1c03800 (PMC8794001; doi:10.1021/acs.chemmater.1c03800)
Supplement: Supplementary file 1 — cm1c03800_si_001.pdf [file cm1c03800_si_001.pdf]

Supporting Information

Indium(II) chloride as a Precursor in the Synthesis of Ternary (Ag-In-S) and Quaternary (Ag-In-Zn-S) Nanocrystals

Patrycja Kowalik,[a,b] Piotr Bujak,[a]* Mateusz Penkala,[c] Anna M. Maroń,[c] Andrzej Ostrowski,[a] Angelika Kmita,[d] Marta Gajewska,[d] Wojciech Lisowski,[e] Janusz W. Sobczak[e] and Adam Pron[a]*

[a]*Faculty of Chemistry, Warsaw University of Technology, Noakowskiego 3, 00-664 Warsaw, Poland, E-mail: piotrbujakchem@poczta.onet.pl; apron@ch.pw.edu.pl*

[b]*Faculty of Chemistry, University of Warsaw, Pasteura 1 Str., PL-02-093 Warsaw, Poland*

[c]*Institute of Chemistry, Faculty of Science and Technology, University of Silesia, Szkolna 9, 40-007 Katowice, Poland*

[d]*Academic Centre for Materials and* Nanotechnology, *AGH University of Science and Technology, , al. Mickiewicza 30, 30-059 Krakow, Poland*

[e]*Institute of Physical Chemistry, Polish Academy of Science, Kasprzaka 44/52, 01-224 Warsaw, Poland*

Experimental

Characterization Methods. Elemental analysis was carried out with a multichannel Quantax 400 energy-dispersive X-ray spectroscopy (EDS) system with a 125 eV xFlash detector 5010 (Bruker) using a 15 kV electron beam energy. X-ray powder diffractograms were recorded at room temperature on a Bruker D8 Advance diffractometer equipped with a LYNXEYE position-sensitive detector using Cu Kα radiation (λ = 0.15418 nm). The data were collected in the Bragg-Brentano ($\theta/2\theta$) horizontal geometry (flat reflection mode) between 10° and 70° (2θ) in a continuous scan, using 0.04° steps at 960 s/step. The incident-beam path in the diffractometer was equipped with a 2.5° Soller slit and a 1.14° fixed divergence slit, whereas the path of the diffracted beam was equipped with a programmable antiscatter slit (fixed at 2.20°), a Ni β-filter, and a 2.5° Soller slit. The sample holder was rotated at an angular speed of 15 rpm. The data were collected under standard conditions (temperature and relative humidity). For XPS analysis the nanocrystals were first dispersed in chloroform, then deposited on a Si(100) wafer and dried at room temperature. Survey and high-resolution (HR) XPS spectra were recorded using a PHI 5000 VersaProbe™ (ULVAC-PHI) spectrometer with monochromatic Al Kα radiation (hν = 1486.6 eV). The HR XPS spectra were collected with the hemispherical analyzer at the pass energy of 23.5 eV, the energy step size of 0.1 eV and the photoelectron take off angle of 45° with respect to the surface plane. The CasaXPS software was used to evaluate the obtained XPS data. Deconvolution of HR XPS spectra were performed using a Shirley background and a Gaussian peak shape with 30% Lorentzian character. The binding energy (BE) scale of all detected spectra was referenced by setting the BE of C1s to 284.8eV. For quantification the PHI Multipak sensitivity factors and determined transmission function of the spectrometer were used. Transmission electron microscopy (TEM) analysis was performed on a Zeiss Libra 120 electron microscope operating at 120 kV. High-resolution images were acquired by a Tecnai TF20 X-TWIN (Thermo Fisher Scientific) microscope operated at 200 kV. ^{1}H, ^{1}H-^{1}H COSY and ^{1}H-^{13}C HMBC NMR spectra were recorded on a Bruker Avance (400 MHz) spectrometer and referenced with respect to tetramethylsilane (TMS) and solvents. UV-vis-NIR spectra were registered using a Cary 5000 (Varian) spectrometer. Steady-state PL spectra of toluene solutions of samples were measured with FLS-980 fluorescence spectrophotometer equipped with a 450 W Xe lamp and photomultiplier (Hamamatsu, R928P) detector with a standard 10 mm cuvette (λ_{exc} = 375 nm).[1] The quantum yields were examined using integrating sphere absolute method with solvent used as blank. The compounds were excited with wavelengths in each case corresponding to the obtained

excitations. The emission correction file was used to take into account the sensitivity of the monochromator, detector, sphere coating and optics to wavelength. Each scans were conducted with 0.25 nm steps, 0.2 Dwell time as well as repeated 2 times. The FLS-980 software was used to designate the quantum yield values. The PL lifetime measurement was performed with a time correlated single photon counting (TCSPC) method on FLS-980 fluorescence spectrophotometer. The excitation wavelength (405 nm) was obtained using the picosecond pulsed diode laser (EPL - 405 nm, Edinburgh Instruments). The system was aligned at the emission wavelengths. Additionally, for the analysis of a fluorescence decay lower than 50ns, an instrument response function (IRF) was obtained. The IRF contains the information about the time response of the overall optical and electronic system. The IRF was designated using solutions of LUDOX® as a standard at excitation wavelength. The decay components were evaluated using the FLS-980 software.

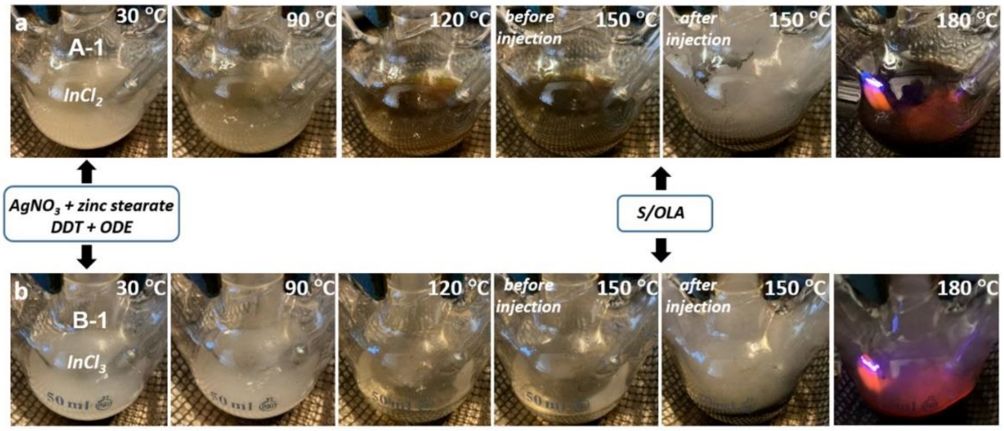

Figure S1. Photographs of the reaction mixtures changing color, due to the addition of a different indium precursors: $InCl_2$ (**A-1**) (a) and $InCl_3$ (**B-1**) (b).

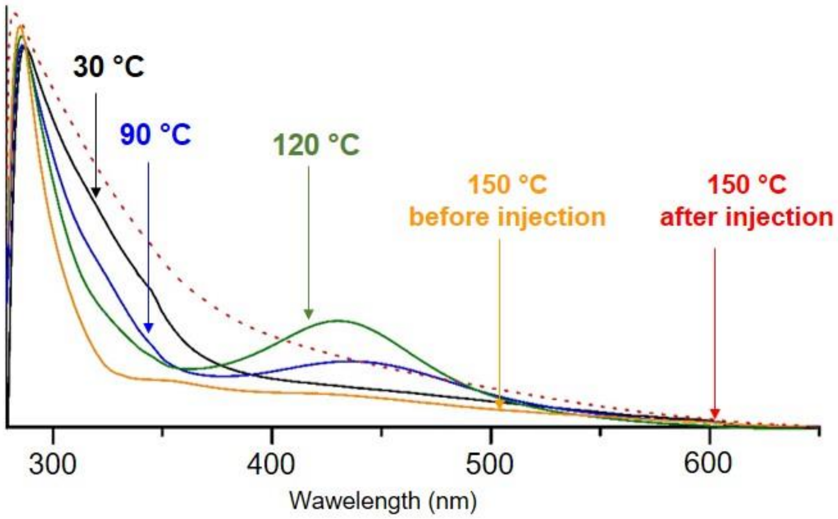

Figure S2. Evolution of UV-vis spectra of the reaction mixture containing indium(II) chloride (batch **A-1**) before and after injection of the sulfur precursor.

The spectra were obtained by sampling the reaction mixture used for the preparation of Ag-In-Zn-S nanocrystals (batch **A-1**) at different stages of its heating before and after injection of the sulfur precursor. The observed changes can be interpreted as follows:

During heating in the temperature range from 30 to 120 °C an intermediate reaction product is formed as evidenced by the transformation of a featureless spectral line into a clear spectrum characterized by a distinct absorption band with a maximum at *ca*. 430 nm. This intermediate product is then decomposed at higher temperature since its spectrum vanishes at 150°C, prior to the injection of the precursor of sulfur.

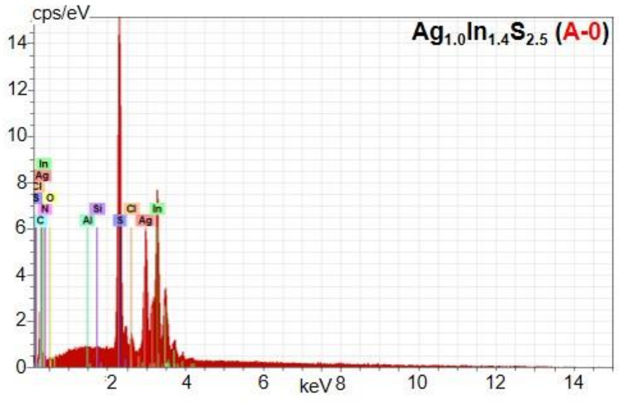

Element	Series	unn. C [wt.%]	norm. C [wt.%]	Atom. C [at.%]	Error (1 Sigma) [wt.%]
Carbon	K-series	6.08	6.92	29.53	0.97
Nitrogen	K-series	0.30	0.34	1.26	0.14
Oxygen	K-series	0.51	0.58	1.85	0.19
Aluminium	K-series	0.03	0.04	0.07	0.03
Silicon	K-series	0.03	0.04	0.07	0.03
Sulfur	K-series	18.44	20.98	33.54	0.68
Chlorine	K-series	1.00	1.14	1.65	0.07
Silver	L-series	24.42	27.78	13.20	0.80
Indium	L-series	37.08	42.18	18.83	1.17
	Total:	87.90	100.00	100.00	

Figure S3. Energy-dispersive spectrum of $\mathbf{Ag_{1.0}In_{1.4}S_{2.5}}$ (**AIS**) nanocrystals.

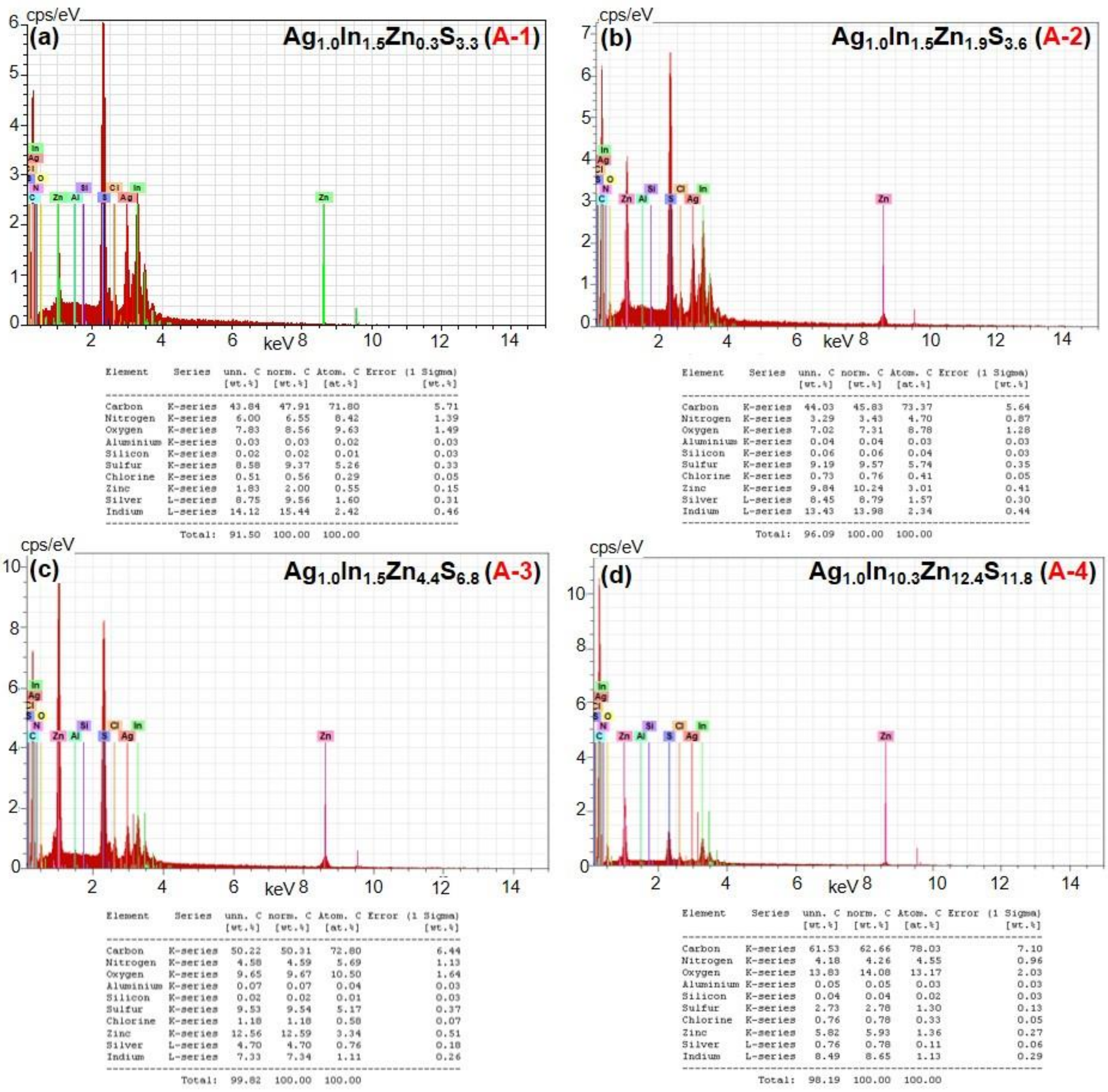

Element	Series	unn. C [wt.%]	norm. C [wt.%]	Atom. C [at.%]	Error (1 Sigma) [wt.%]
Carbon	K-series	43.84	47.91	71.80	5.71
Nitrogen	K-series	6.00	6.55	8.42	1.39
Oxygen	K-series	7.83	8.56	9.63	1.49
Aluminium	K-series	0.03	0.03	0.02	0.03
Silicon	K-series	0.02	0.02	0.01	0.03
Sulfur	K-series	8.58	9.37	5.26	0.33
Chlorine	K-series	0.51	0.56	0.29	0.05
Zinc	K-series	1.83	2.00	0.55	0.15
Silver	L-series	8.75	9.56	1.60	0.31
Indium	L-series	14.12	15.44	2.42	0.46
	Total:	91.50	100.00	100.00	

Element	Series	unn. C [wt.%]	norm. C [wt.%]	Atom. C [at.%]	Error (1 Sigma) [wt.%]
Carbon	K-series	44.03	45.83	73.37	5.64
Nitrogen	K-series	3.29	3.43	4.70	0.87
Oxygen	K-series	7.02	7.31	8.78	1.28
Aluminium	K-series	0.04	0.04	0.03	0.03
Silicon	K-series	0.06	0.06	0.04	0.03
Sulfur	K-series	9.19	9.57	5.74	0.35
Chlorine	K-series	0.73	0.76	0.41	0.05
Zinc	K-series	9.84	10.24	3.01	0.41
Silver	L-series	8.45	8.79	1.57	0.30
Indium	L-series	13.43	13.98	2.34	0.44
	Total:	96.09	100.00	100.00	

Element	Series	unn. C [wt.%]	norm. C [wt.%]	Atom. C [at.%]	Error (1 Sigma) [wt.%]
Carbon	K-series	50.22	50.31	72.80	6.44
Nitrogen	K-series	4.58	4.59	5.69	1.13
Oxygen	K-series	9.65	9.67	10.50	1.64
Aluminium	K-series	0.07	0.07	0.04	0.03
Silicon	K-series	0.02	0.02	0.01	0.03
Sulfur	K-series	9.53	9.54	5.17	0.37
Chlorine	K-series	1.18	1.18	0.58	0.07
Zinc	K-series	12.56	12.59	3.34	0.51
Silver	L-series	4.70	4.70	0.76	0.18
Indium	L-series	7.33	7.34	1.11	0.26
	Total:	99.82	100.00	100.00	

Element	Series	unn. C [wt.%]	norm. C [wt.%]	Atom. C [at.%]	Error (1 Sigma) [wt.%]
Carbon	K-series	61.53	62.66	78.03	7.10
Nitrogen	K-series	4.18	4.26	4.55	0.96
Oxygen	K-series	13.83	14.08	13.17	2.03
Aluminium	K-series	0.05	0.05	0.03	0.03
Silicon	K-series	0.04	0.04	0.02	0.03
Sulfur	K-series	2.73	2.78	1.30	0.13
Chlorine	K-series	0.76	0.78	0.33	0.05
Zinc	K-series	5.82	5.93	1.36	0.27
Silver	L-series	0.76	0.78	0.11	0.06
Indium	L-series	8.49	8.65	1.13	0.29
	Total:	98.19	100.00	100.00	

Figure S4. Energy-dispersive spectra of $\mathbf{Ag_{1.0}In_{1.5}Zn_{0.3}S_{3.3}}$ (**A-1**) (a), $\mathbf{Ag_{1.0}In_{1.5}Zn_{1.9}S_{3.6}}$ (**A-2**) (b), $\mathbf{Ag_{1.0}In_{1.5}Zn_{4.4}S_{6.8}}$ (**A-3**) (c) and $\mathbf{Ag_{1.0}In_{10.3}Zn_{12.4}S_{11.8}}$ (**A-4**) (d) alloyed nanocrystals.

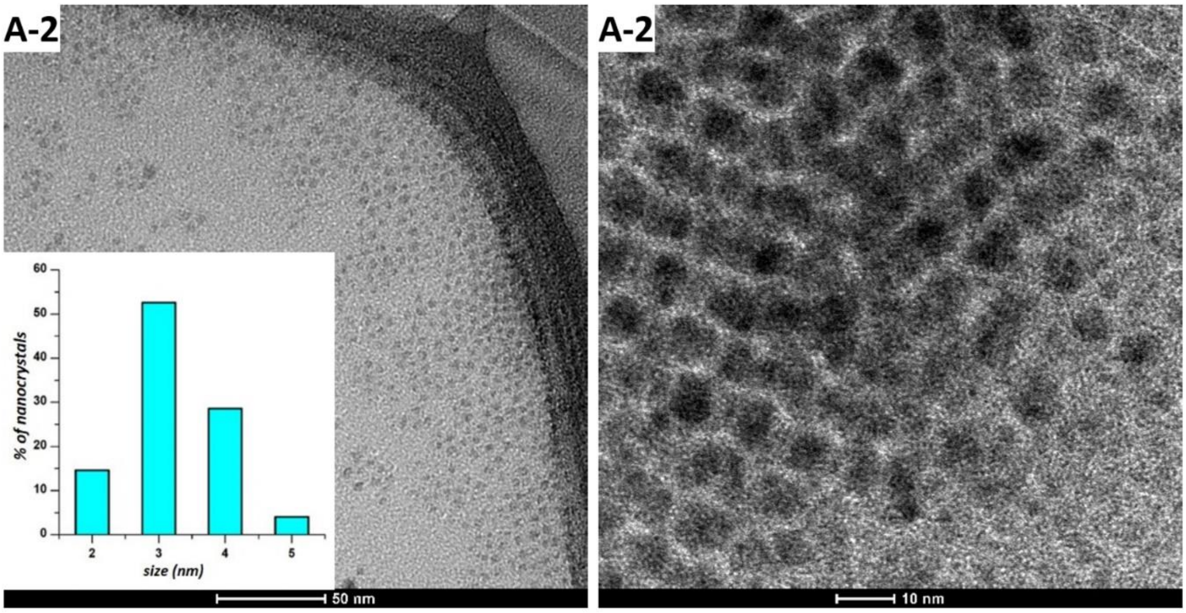

Figure S5. TEM and HR-TEM images of **$Ag_{1.0}In_{1.5}Zn_{1.9}S_{3.6}(S_{4.6})$** (**A-2**) alloyed nanocrystals and their corresponding histogram.

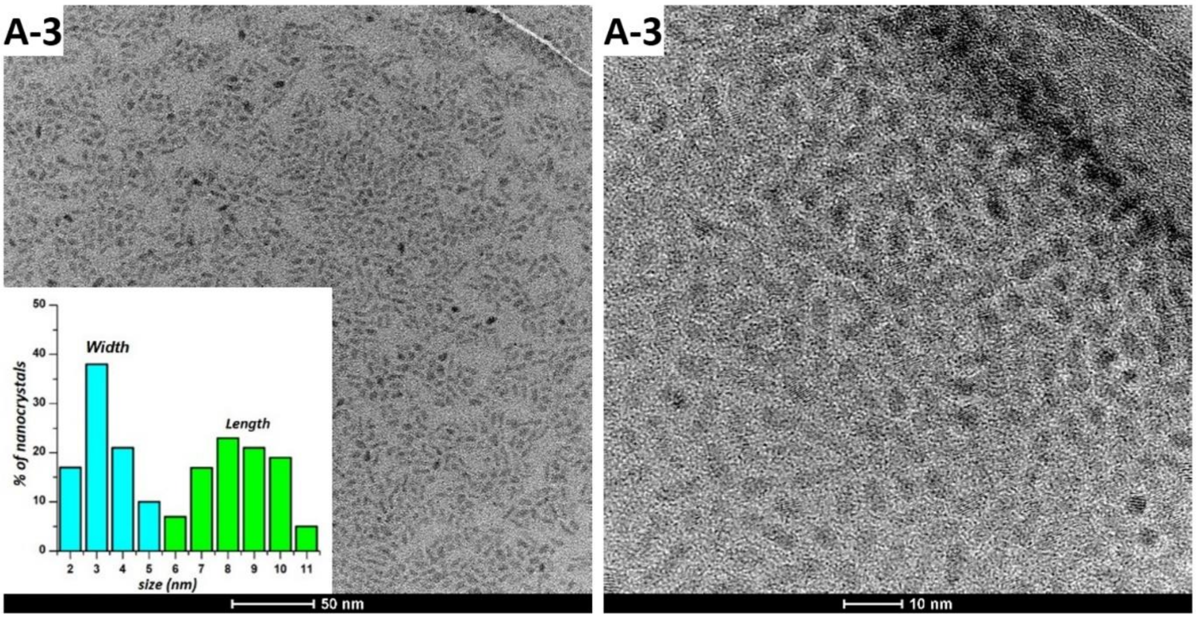

Figure S6. TEM and HR-TEM images of **$Ag_{1.0}In_{1.5}Zn_{4.4}S_{6.8}(S_{7.1})$** (**A-3**) alloyed nanocrystals and their corresponding histogram.

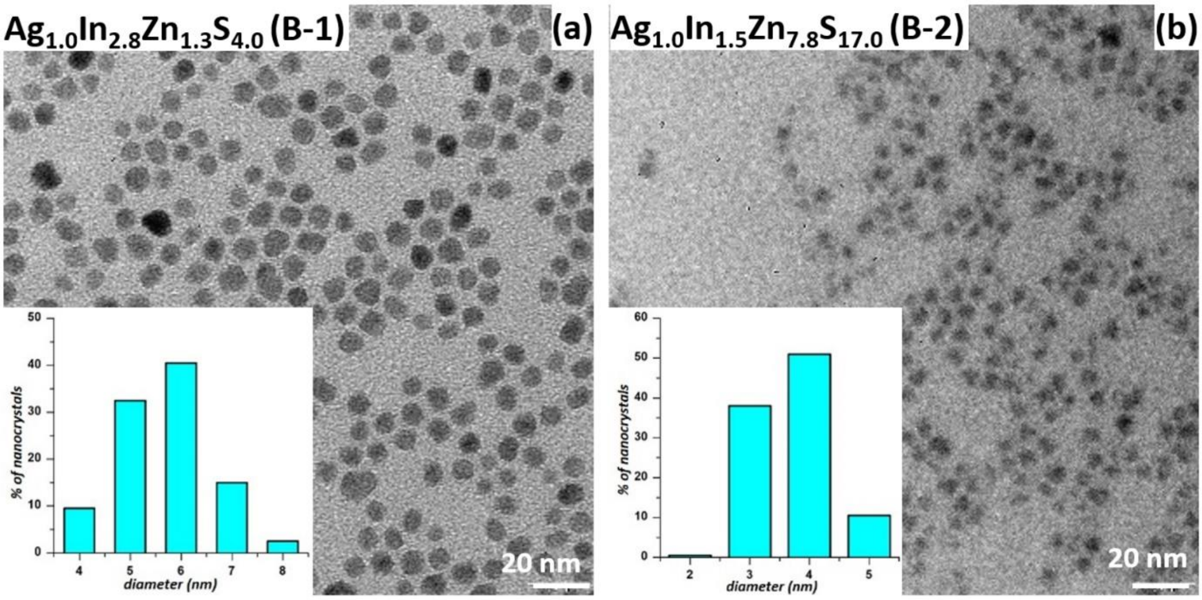

Figure S7. TEM images of **$Ag_{1.0}In_{2.8}Zn_{1.3}S_{4.0}(S_{6.0})$** (**B-1**, D = 6.2 ±0.9 nm) (a) and **$Ag_{1.0}In_{1.5}Zn_{7.8}S_{17.0}(S_{10.5})$** (**B-2**, D = 3.3 ±0.9 nm) (b) alloyed nanocrystals and their corresponding histograms.[2]

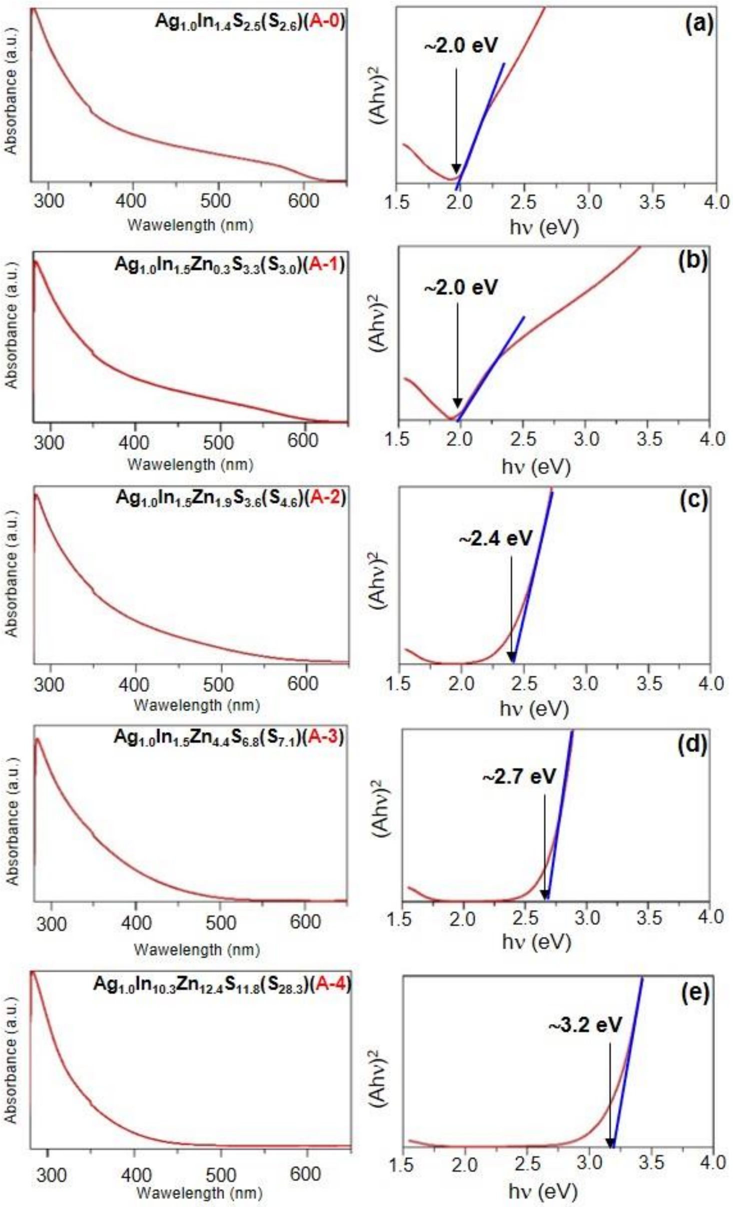

Figure S8. Room temperature UV-vis-NIR spectra of toluene dispersion of $\mathbf{Ag_{1.0}In_{1.4}S_{2.5}(S_{2.6})}$ (**AIS**) (a) and alloyed $\mathbf{Ag_{1.0}In_{1.5}Zn_{0.3}S_{3.3}(S_{3.0})}$ (**A-1**) (b), $\mathbf{Ag_{1.0}In_{1.5}Zn_{1.9}S_{3.6}(S_{4.6})}$ (**A-2**) (c), $\mathbf{Ag_{1.0}In_{1.5}Zn_{4.4}S_{6.8}(S_{7.1})}$ (**A-3**) (d), and $\mathbf{Ag_{1.0}In_{10.3}Zn_{12.4}S_{11.8}(S_{28.3})}$ (**A-4**) (e) nanocrystals and the corresponding $(Ah\nu)^2$ *vs* $h\nu$ curves (where A = absorbance, h = Planck's content and ν = frequency).

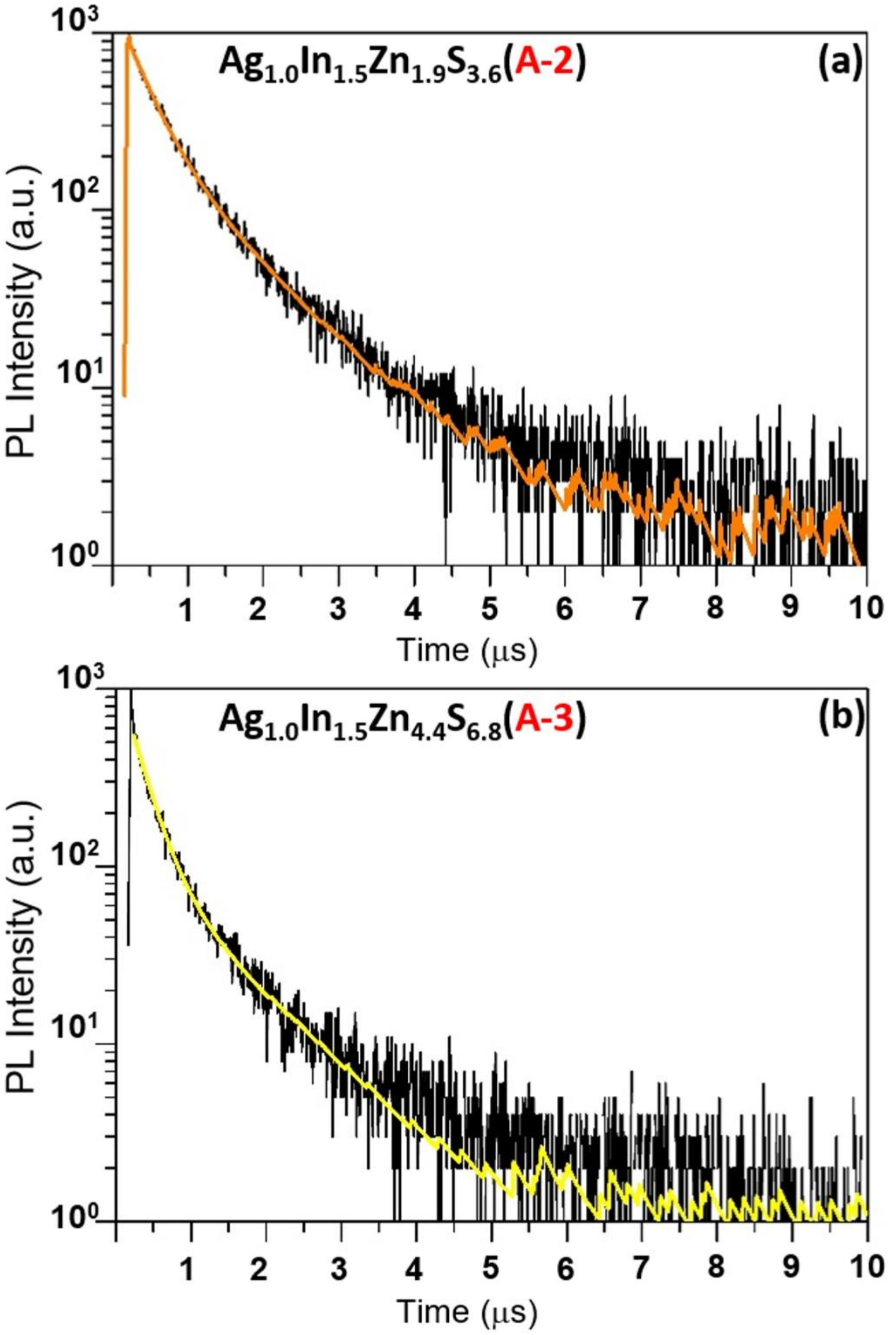

Figure S9. Photoluminescence decay curves of **$Ag_{1.0}In_{1.5}Zn_{1.9}S_{3.6}(S_{4.6})$** (**A-2**) (a) and **$Ag_{1.0}In_{1.5}Zn_{4.4}S_{6.8}(S_{7.1})$** (**A-3**) (b) alloyed nanocrystals and the corresponding fitting curves.

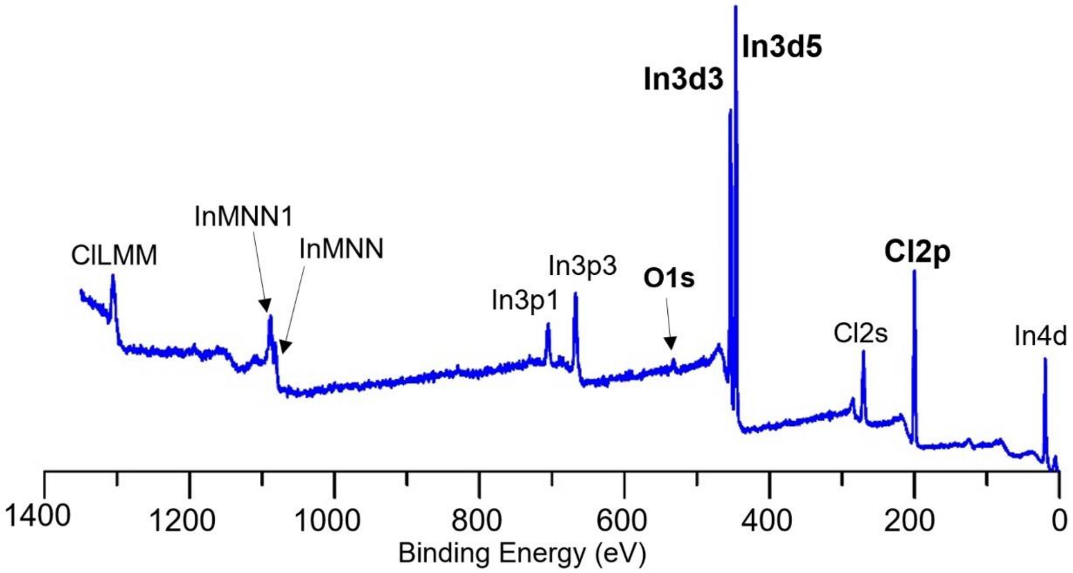

Figure S10. XPS survey spectrum of indium(II) chloride.

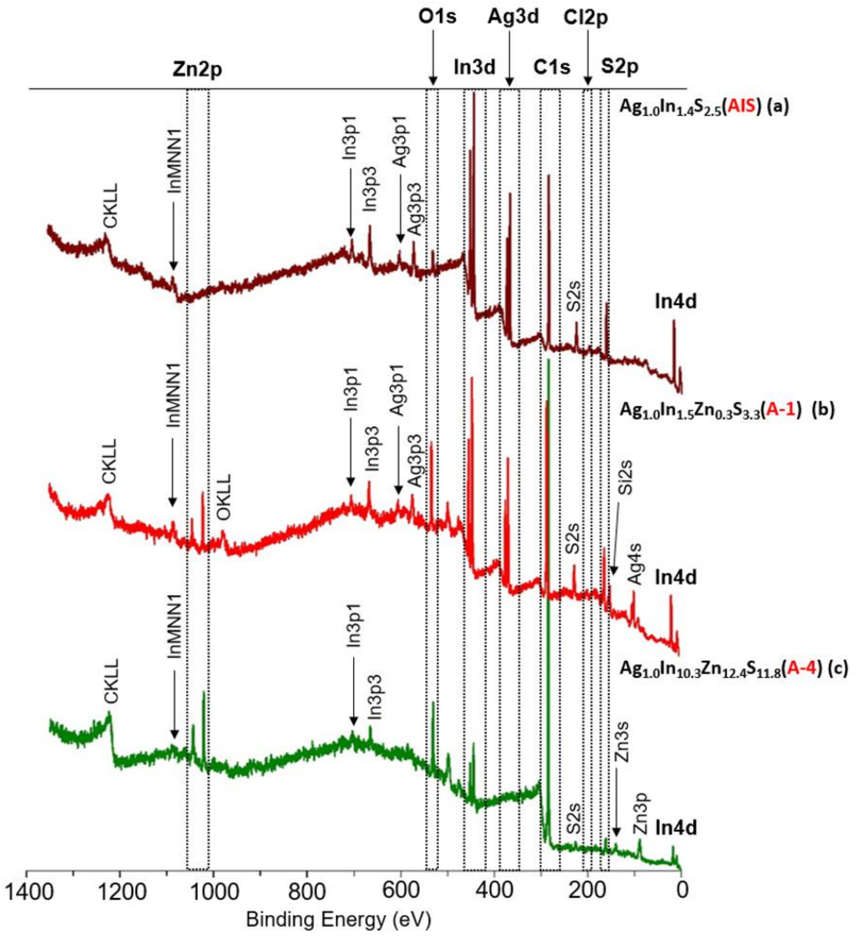

Figure S11. XPS survey spectra of $\mathbf{Ag_{1.0}In_{1.4}S_{2.5}(S_{2.6})}$ (**AIS**) (a), $\mathbf{Ag_{1.0}In_{1.5}Zn_{0.3}S_{3.3}(S_{3.0})}$ (**A-1**) (b) and $\mathbf{Ag_{1.0}In_{10.3}Zn_{12.4}S_{11.8}(S_{28.3})}$ (**A-4**) (c) nanocrystals.

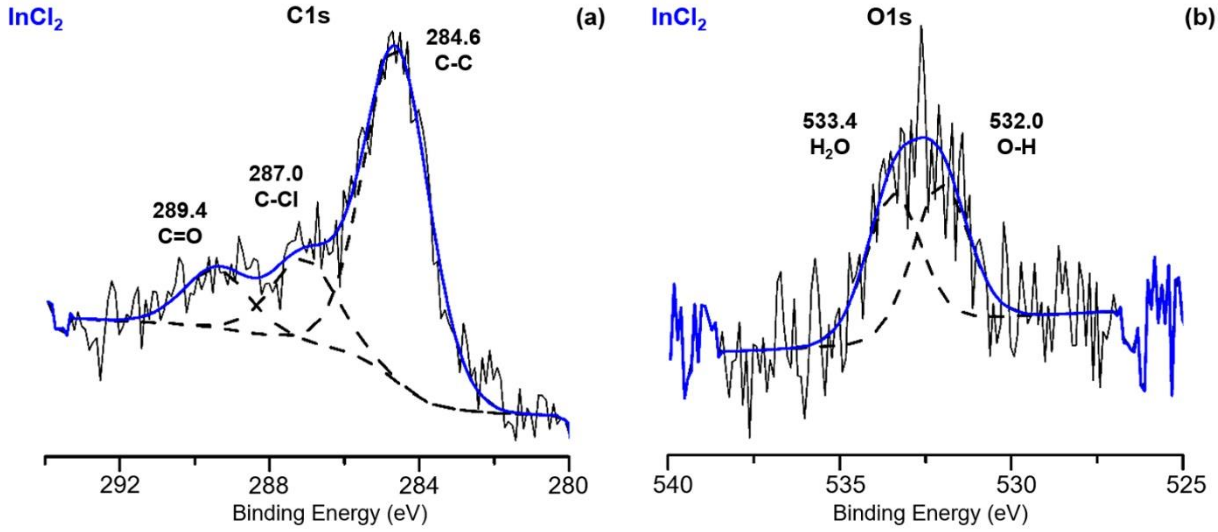

Figure S12. The C1s (a) and O1s (b) high-resolution XPS spectra of indium(II) chloride.

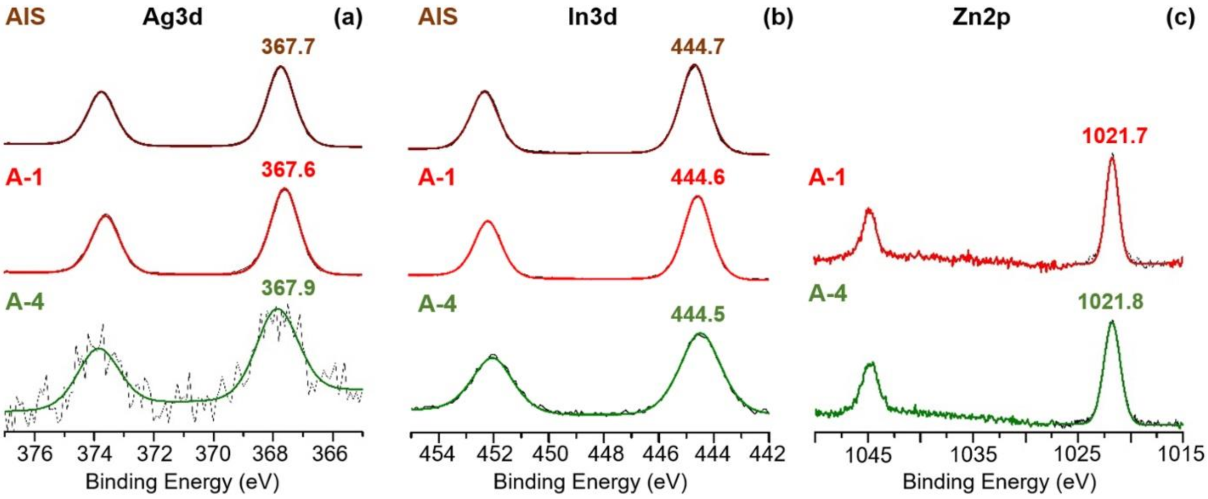

Figure S13. The Ag3d (a), In3d (b) and Zn2p (c) high-resolution XPS spectra of **$Ag_{1.0}In_{1.4}S_{2.5}(S_{2.6})$** (**AIS**), **$Ag_{1.0}In_{1.5}Zn_{0.3}S_{3.3}(S_{3.0})$** (**A-1**) and **$Ag_{1.0}In_{10.3}Zn_{12.4}S_{11.8}(S_{28.3})$** (**A-4**) nanocrystals.

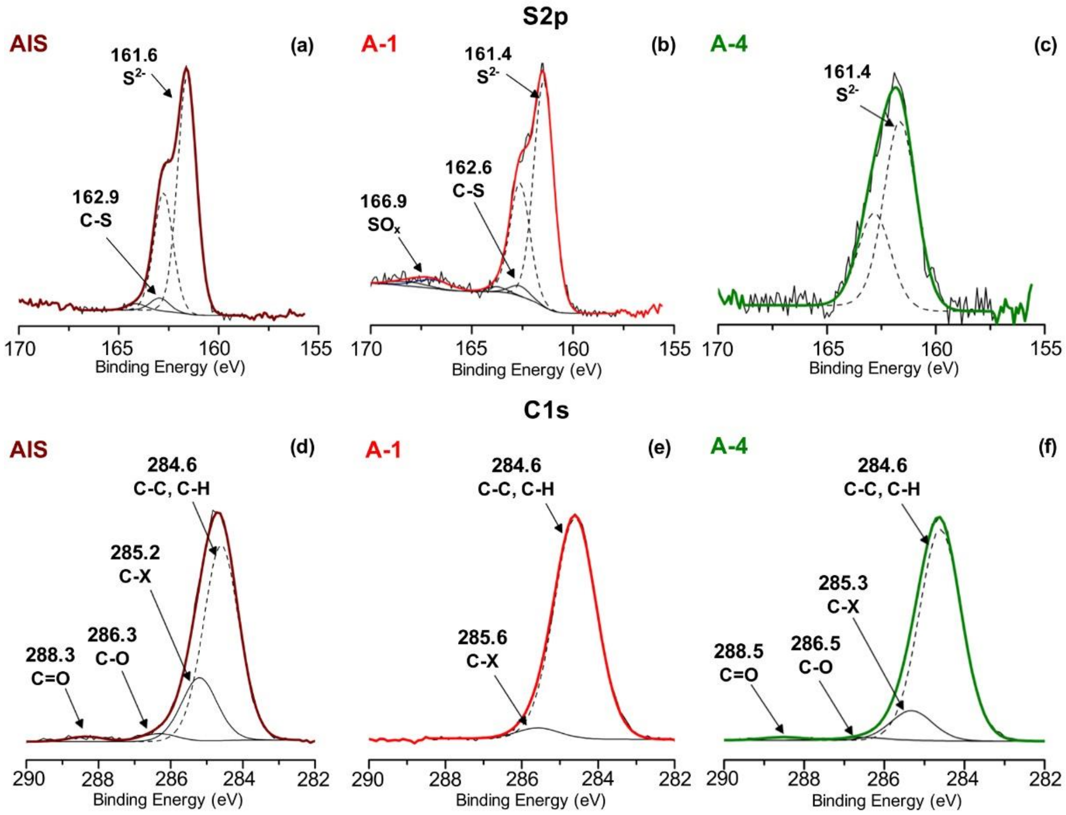

Figure S14. S2p (a-c) and C1s (d-f) high-resolution XPS spectra of **$Ag_{1.0}In_{1.4}S_{2.5}(S_{2.6})$** (**AIS**), **$Ag_{1.0}In_{1.5}Zn_{0.3}S_{3.3}(S_{3.0})$** (**A-1**) and **$Ag_{1.0}In_{10.3}Zn_{12.4}S_{11.8}(S_{28.3})$** (**A-4**) nanocrystals.

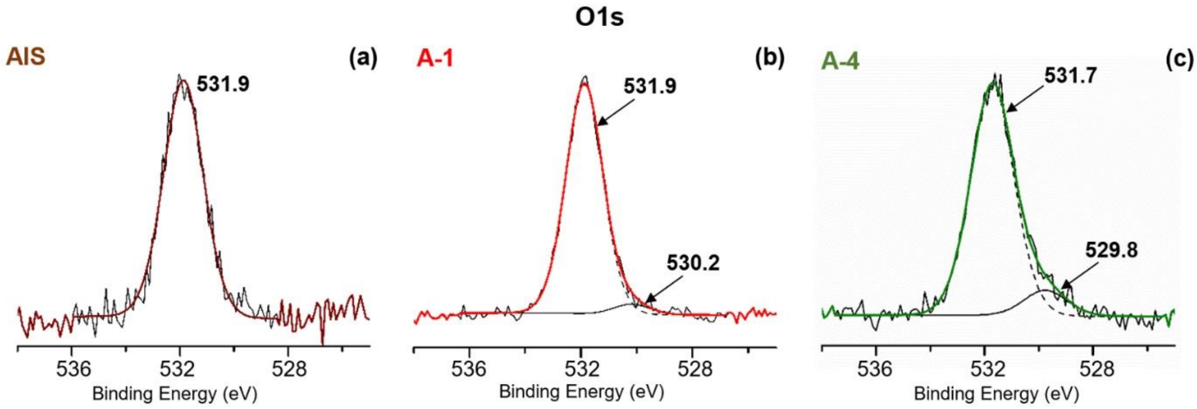

Figure S15. The O1s high-resolution XPS spectra of $\mathbf{Ag_{1.0}In_{1.4}S_{2.5}(S_{2.6})}$ (**AIS**), $\mathbf{Ag_{1.0}In_{1.5}Zn_{0.3}S_{3.3}(S_{3.0})}$ (**A-1**) and $\mathbf{Ag_{1.0}In_{10.3}Zn_{12.4}S_{11.8}(S_{28.3})}$ (**A-4**) nanocrystals.

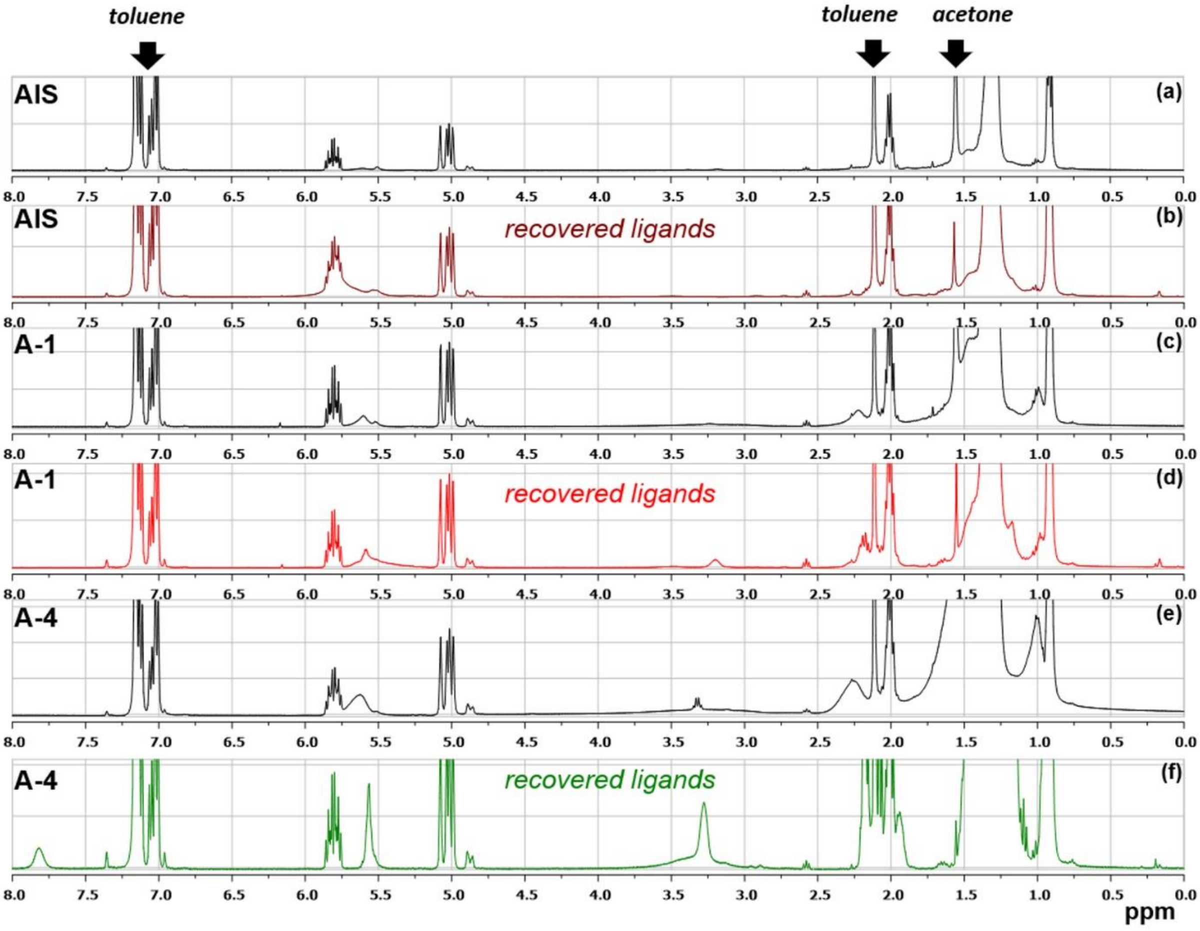

Figure S16. ¹H NMR spectra of C_6D_6 dispersion of $\mathbf{Ag_{1.0}In_{1.4}S_{2.5}(S_{2.6})}$ (**AIS**) (a), $\mathbf{Ag_{1.0}In_{1.5}Zn_{0.3}S_{3.3}(S_{3.0})}$ (**A-1**) (c), $\mathbf{Ag_{1.0}In_{10.3}Zn_{12.4}S_{11.8}(S_{28.3})}$ (**A-4**) (e) nanocrystals capped with initial ligands and ligands recovered after dissolution of nanocrystals (b, d and f).

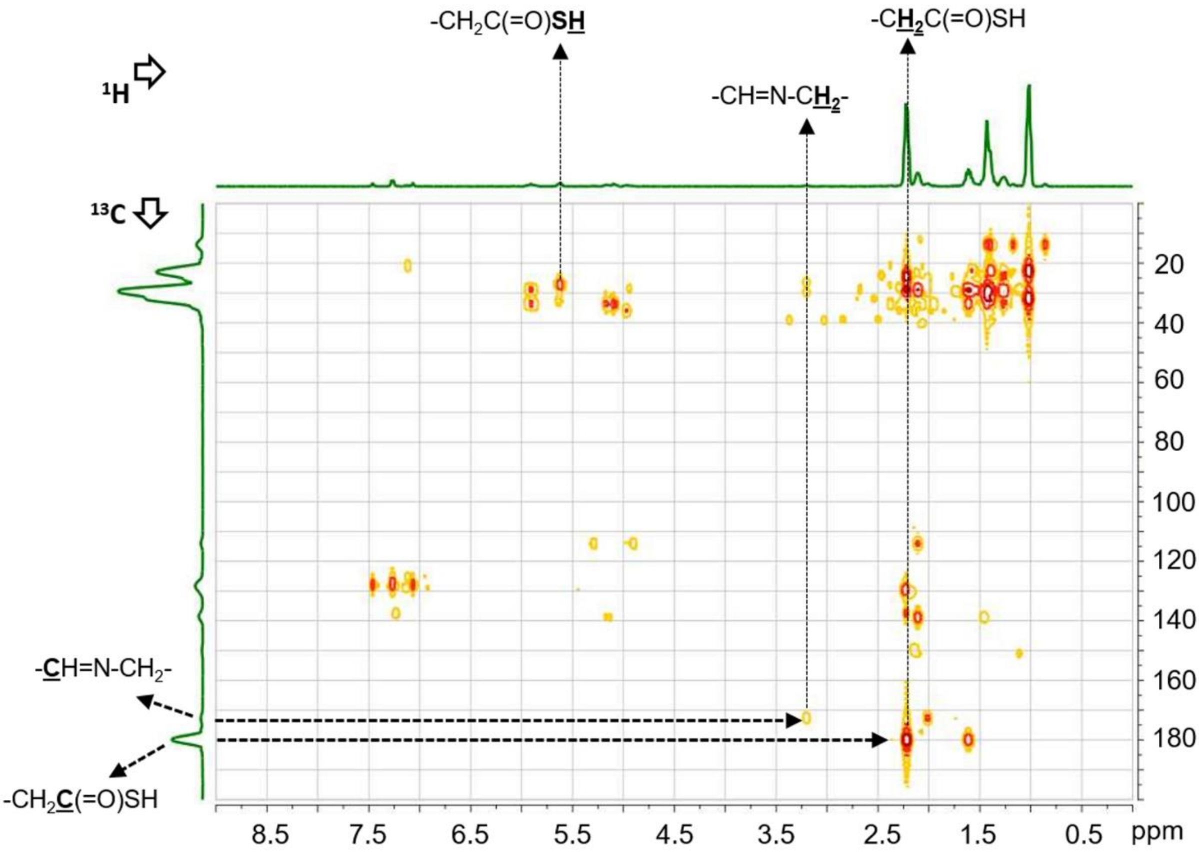

Figure S17. ^1H-^{13}C HMBC NMR spectrum of the organic residue from $\mathbf{Ag_{1.0}In_{10.3}Zn_{12.4}S_{11.8}(S_{28.3})}$ (**A-4**) nanocrystals recorded in C_6D_6.

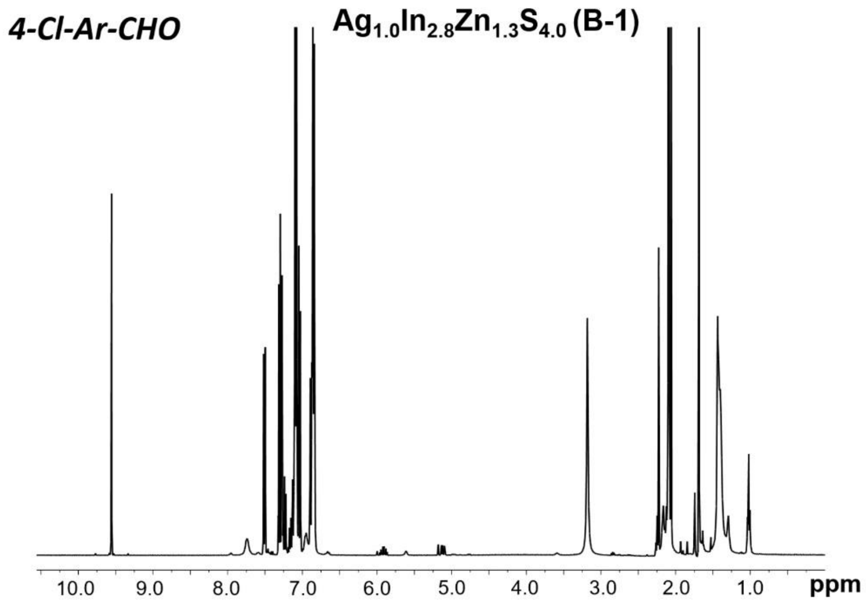

Figure S18. ^1H NMR spectrum of the photocatalytic reaction mixture used for photocatalytic reduction of 4-benzaldehyde with **B-1** ($\mathbf{Ag_{1.0}In_{2.8}Zn_{1.3}S_{4.0}(S_{6.0})}$) nanocrystals as photocatalyst (in C_6D_6).

Table S1. Comparison of the photoluminescence properties (maxima of the photoluminescence band (PL), quantum yields (Q.Y.), three- and biexponential fit parameters and average emission lifetimes) of $AgInS_2$ and $AgInS_2$-ZnS nanocrystals.

Reference	Materials	Ligands	λ [nm]	Q.Y. [%]	τ_1 [ns]	A_1 [%]	τ_2 [ns]	A_2 [%]	τ_3 [ns]	A_3 [%]	τ_{av} [ns]
This work	$AgIn_{1.4}S_{2.5}$	Hydrophobic	755	12	1151	83.4	13120	16.6	-	-	9458
	$AgIn_{1.5}Zn_{0.3}S_{3.3}$ alloy	Hydrophobic	731	40	139	16.5	591	50.7	1921	32.8	1460
2012 [3]	$AgIn_{2.3}S_{3.9}$	Hydrophobic	725	25	440	83	1200	17	-	-	712
2012 [4]	$AgInZn_{2.4}$	Hydrophobic	520	41	79	16.8	441	83.1	-	-	428
2014 [5]	$AgInZn_{3.9}$	Hydrophobic	650	44	280	47.4	1248	52.6	-	-	1085
2015 [6]	$AgInS_2$	Hydrophilic	817	34	115	-	853	-	-	-	-
2016 [7]	$AgInS_{2.3}$	Hydrophobic	600	7	47	57	316	43	-	-	271
	$AgIn_{1.3}Zn_{1.3}S_{4.7}$ core/shell	Hydrophobic	550	40	54	40	351	60	-	-	323
2018 [8]	$AgIn_{2.3}Zn_{0.6}S_{2.5}$ alloy	Hydrophilic	550	41	31	11	266	89	-	-	262
2018 [9]	$AgInS_2$	Hydrophilic	600	22	66	24	390	76	-	-	374
2018 [10]	$AgIn_{1.2}Zn_{1.3}S$ alloy	Hydrophobic	650	38	9	46	670	39	2400	16	1700
2020 [11]	$AgIn_{1.9}S_2$	Hydrophilic	710	36	26	1	160	13	813	86	794
	$AgIn_{2.9}Zn_{1.7}S_2/(ZnS)_{5.0}$ core/shell	Hydrophilic	696	55	290	24	936	76	-	-	878
2020 [12]	$AgInS_2$	Hydrophilic	572	37	1160	95	9630	5	-	-	3736

References

(1) Maron, A. M.; Szłapa-Kula, A.; Matussek, M.; Kruszyński, R.; Siwy, M.; Janeczek, H.; Grzelak, J.; Mackowski, S.; Schab-Balcerzak, E.; Machura, B. Photoluminescence Enhancement of Re(I) Carbonyl Complexes Bearing D-A and D-π-A ligands. *Dalton Trans.* **2020**, *49*, 4441-4453.

(2) Kowalik, P.; Bujak, P.; Penkala, M.; Pron, A. Organic-to-Aqueous Phase Transfer of Alloyed $AgInS_2$-ZnS Nanocrystals Using Simple Hydrophilic Ligands: Comparison of 11-Mercaptoundecanoic Acid, Dihydrolipoic Acid and Cysteine. *Nanomaterials* **2021**, *11*, 843.

(3) Dai, M.; Ogawa, S.; Kameyama, T.; Okazaki, K.-i.; Kudo, A.; Kuwabata, S.; Tsuboi, Y.; Torimoto, T. Tunable Photoluminescence from the Visible to Near-Infrared Wavelength Region of Non-Stoichiometric $AgInS_2$ Nanoparticles. *J. Mater. Chem.* **2012**, *22*, 12851-12858.

(4) Tang, X.; Ho, W. B. A.; Xue, J. M. Synthesis of Zn-Doped $AgInS_2$ Nanocrystals and Their Fluorescence Properties. *J. Phys. Chem. C* **2012**, *116*, 9769-9773.

(5) Tang, X.; Zang, Z.; Zu, Z.; Chen, W.; Liu, Y.; Han, G.; Lei, X.; Liu, X.; Du, X.; Chen, W.; Wang, Y.; Xue, J. A Facile Method for the Synthesis of Quaternary Ag-In-Zn-S Alloyed Nanorods. *Nanoscale* **2014**, *6*, 11803-11809.

(6) Tan, L.; Liu, S.; Li, X.; Chronakis, I. S.; Shen, Y. A New Strategy for Synthesizing $AgInS_2$ Quantum Dots Emitting Brightly in Near-Infrared Window for In Vivo Imaging. *Colloids Surf. B* **2015**, *125*, 222-229.
(7) Chen, S.; Ahmadiantehrani, M.; Zhao, J.; Zhu, S.; Mamalis, A. G.; Zhu, X. Heat-up Synthesis of Ag-In-S and Ag-In-S/ZnS Nanocrystals: Effect of Indium Precursors on their Optical Properties. *J. Alloys Compd.* **2016**, *665*, 137-143.
(8) Liu, Y.; Tang, X.; Deng, M.; Zhu, T.; Bai, Y.; Qu, D.; Huang, X.; Qiu, F. One-Step Aqueous Synthesis of Highly Luminescent Hydrophilic AgInZnS Quantum Dots. *J. Lumin.* **2018**, *202*, 71-76.
(9) Hu, X.; Chen, T.; Xu, Y.; Wang, M.; Jiang, W.; Jiang, W. Hydrothermal Synthesis of Bright and Stable $AgInS_2$ Quantum Dots with Tunable Visible Emission. *J. Lumin.* **2018**, *200*, 189-195.
(10) Kameyama, T.; Koyama, S.; Yamamoto, T.; Kuwabata, S.; Torimoto, T. Enhanced Photocatalytic Activity of Ag-In-Zn-S Semiconductor Nanocrystals with a Dumbbell-Shaped Heterostructure. *J. Phys. Chem. C* **2018**, *122*, 13705-13715.
(11) Delices, A.; Moodelly, D.; Hurot, C.; Hou, Y.; Ling, W. L.; Saint-Pierre, C.; Gasparutto, D.; Nogues, G.; Reiss, P.; Kheng, K. Aqueous Synthesis of DNA-Functionalized Near-Infrared $AgInS_2$/ZnS Core/Shell Quantum Dots. *ACS Appl. Mater. Interfaces* **2020**, *12*, 44026-44038.
(12) Jiao, M.; Li, Y.; Jia, Y.; Li, C.; Bian, H.; Gao, L.; Cai, P.; Luo, X. Strongly Emitting and Long-Lived Silver Indium Sulfide Quantum Dots for Bioimaging: Insight into co-Ligand Effect on Enhanced Photoluminescence. *J. Colloid Interface Sci.* **2020**, *565*, 35-42.
